# Effects of mouth breathing on facial skeletal development in children: a systematic review and meta-analysis

**DOI:** 10.1186/s12903-021-01458-7

**Published:** 2021-03-10

**Authors:** Ziyi Zhao, Leilei Zheng, Xiaoya Huang, Caiyu Li, Jing Liu, Yun Hu

**Affiliations:** 1 The Affiliated Stomatology Hospital of Chongqing Medical University, No.426 Songshi North Road, Yubei district, Chongqing, 401147 China; 2 Chongqing Key Laboratory of Oral Diseases and Biomedical Sciences, Chongqing, 401147 China; 3 Chongqing Municipal Key Laboratory of Oral Biomedical Engineering of Higher Education, Chongqing, 401147 China

**Keywords:** Mouth breathing, Facial skeletal development, Children, Systematic review, Meta-analysis

## Abstract

**Background:**

Mouth breathing is closely related to the facial skeletal development and malocclusion. The purpose of this systematic review and meta-analysis was to assess the effect of mouth breathing on facial skeletal development and malocclusion in children.

**Methods:**

An electronic search in PubMed, the Cochrane Library, Medline, Web of Science, EMBASE and Sigle through February 23rd, 2020, was conducted. Inclusion criteria were children under 18 years of age with maxillofacial deformities due to mouth breathing. The risk of bias in nonrandomized studies of interventions (ROBINS-I) tool for controlled clinical trials. The Grading of Recommendation, Assessment, Development and Evaluation (GRADE) approach was used for the quality assessment. The included indicators were SNA, SNB, ANB, SN-OP, SN-PP, PP-MP, SNGoGn, MP-H, 1-NA, 1. NA, 1. NB, 1-NB, Overjet, Overbite, SPAS, PAS, and C3-H. Data concerning the mean difference in mesial molar movement and extent of canine retraction were extracted for statistical analysis. The mean differences and 95% confidence intervals were analyzed for continuous data. Review Manager 5.3, was used to synthesize various parameters associated with the impact of mouth breathing on facial skeletal development and malocclusion.

**Results:**

Following full-text evaluations for eligibility, 10 studies were included in the final quantitative synthesis. In Sagittal direction, SNA (MD: − 1.63, *P* < 0.0001), SNB (MD: − 1.96, *P* < 0.0001) in mouth-breathing children was lower than that in nasal-breathing children. ANB (MD: 0.90, *P* < 0.0001), 1. NA (MD: 1.96, *P* = 0.009), 1-NA (MD: 0.66, *P* = 0.004), and 1-NB (MD: 1.03, *P* < 0.0001) showed higher values in children with mouth breathing. In vertical direction, SN-PP (MD: 0.68, *P* = 0.0050), SN-OP (MD: 3.05, *P* < 0.0001), PP-MP (MD: 4.92, *P* < 0.0001) and SNGoGn (MD: 4.10, *P* < 0.0001) were higher in mouth-breathing individuals. In airway, SPAS (MD: − 3.48, *P* = 0.0009), PAS (MD: − 2.11, *P* < 0.0001), and C3-H (MD: − 1.34, *P* < 0.0001) were lower in mouth breathing group.

**Conclusions:**

The results showed that the mandible and maxilla rotated backward and downward, and the occlusal plane was steep. In addition, mouth breathing presented a tendency of labial inclination of the upper anterior teeth. Airway stenosis was common in mouth-breathing children.

*Trial registration* crd-register@york.ac.uk, registration number CRD42019129198.

**Supplementary Information:**

The online version contains supplementary material available at 10.1186/s12903-021-01458-7.

## Background

Mouth breathing is a form of breathing that replaces nasal breathing and it’s aetiology is complex. Mouth breathing may due to genetic factors, poor oral habits, or nasal obstruction, including but not limited to adenoid/tonsil hypertrophy, nasal polyps, nasal septum deviation, turbinate hypertrophy, or sinusitis [1–6]. In addition, mouth breathing may be related to respiratory allergies, climatic conditions, a poor sleeping position, breastfeeding [7].

Currently, the influence of mouth breathing on the development of oral maxillofacial bone is still controversial. Children with mouth breathing often have "adenoid faces" [8], which are characterized as having upper lip incompetence, a retropositioned hyoid bone, a narrow upper dental arch, retropositioned mandibular incisors, an increased anterior face height, a narrow or “V”-shaped maxillary arch, an increased mandibular plane angle, and a posterior-rotated mandible in comparison with healthy controls [9, 10]. With respect to the occlusal relationship, most of the children with mouth breathing presented with Class II malocclusion, and a cross-bite is more frequent than that in those with normal nasal breathing [11]. However, different scholars have reported different research results on the effects of mouth breathing on the maxilla and mandible and the position of the maxilla relative to the skull base. Some scholars believe that mouth breathers’ maxilla was more retrognathic and their anterior lower height of the face was increased, while others have the opposite opinion [12–15]. A growing number of scholars believe that facial skeletal development is greatly improved after the aetiology of mouth breathing is removed by surgery or other means [16–18]. To date, systematic reviews about the effect of mouth breathing on maxillofacial development and malocclusion have been mainly divided into two categories: reviews on the effects of adenoid/tonsil hypertrophy on oral and maxillofacial development before and after oral respiratory surgery and qualitative analyses of the effects of mouth breathing on the occlusal relationship in children. To the best of our knowledge, our study is the first quantitative analysis to explore the effects of mouth breathing on facial bone development and malocclusion in children.

The purpose of this study was to elucidate, through a systematic review and meta-analysis, the changes in facial skeletal development and malocclusion in mouth-breathing children.

## Materials and methods

The format for this systematic review and meta-analysis was based on the Preferred Reporting Items for Systematic Reviews and Meta-Analyses (PRISMA) statement [19]. The PRISMA checklist is shown in Additional file 1: Appendix A1. The inclusion criteria and methods of analysis have been previously specified and documented in a protocol in the PROSPERO database (crd-register@york.ac.uk; registration number CRD42019129198).

### Search strategy

Electronic searches in the PubMed, Cochrane Library, Medline, Web of Science, EMBASE and Sigle databases through February 23rd, 2020, were conducted. There were no language restrictions. The following MeSH terms and texts in various combinations were used: malocclusion, mouth breathing, mandible, maxilla, dentofacial growth, and facial growth (electronic search strategy for PubMed is shown in Additional file 2: Appendix A2). In addition, the references of relevant studies were also searched manually. Two authors (Ziyi Zhao and Leilei Zheng) were trained on the inclusion and exclusion criteria before screening, and pre-screening was conducted to unify the standards in controversial areas. After completing the relevant training, the two authors (Ziyi Zhao and Leilei Zheng) independently screened the study titles and abstracts to identify any potentially eligible studies; then, full-texts were strictly screened according to the inclusion and exclusion criteria. If there was any discrepancy regarding the eligibility of an article, consensus was reached with the guidance of the senior author (Yun Hu).

### Study selection

#### Inclusion criteria

The search strategy was defined according to the patients, exposure, control, outcomes, and study design (PECOS) format: (1) Population: children under the age of 18 with mouth breathing habits; (2) Exposure: mouth breathing due to several causes, including but not limited to tonsil and adenoid hypertrophy, polyps, allergies, recurrent infections and nasal deformities [20]; (3) Control: patients without mouth breathing; (4) Outcome: defects in development in facial bone or dental, which can be embodied in the following cephalometric indicators: SNA, SNB, ANB, PP-MP, SN-MP, SN-PP, SN-OP, OP-MP, FMA, N-Me, SN-Gn, SNGoGn, GoGn, ArGoMe, ArGo, N-ANS, ANS-Me, S-Go, MP-H, 1-NA, 1. NA, 1. NB, 1-NB, SPAS, PAS, C3-H, overbite, and overjet; and (5) Study design: Clinical controlled trials, randomized controlled trials, and cohort studies.

#### Exclusion criteria

The exclusion criteria were as follows: studies that were opinion articles, letters, news reports, editorials, bibliographies, conference summaries, project presentations, data compilation, reviews (although the reviews were not included in this study, related reviews were tracked the original studies according to references) [17, 18]; studies that included children with systemic diseases, lip or palate cleft, oral or maxillofacial trauma or surgical history, orthodontic treatment history and children aged over 18 years.

### Data extraction

The data extracted from the included studies were as follows: the first author's name, year of publication, exposure, sample size, characteristics of the subjects, age of the subjects, and cephalometric outcomes. The cephalometric value data of different groups in the same study were extracted. However, only the original data of the oral and nasal breathing groups before the change in respiratory patterns without treatment or by other means were considered. Unless the same parameters originated from at least two of the selected studies, the relevant data could be described but not synthesized.

### Quality assessment

The risk of bias in nonrandomized studies of interventions (ROBINS-I) tool was used for controlled clinical trials (CCTs) [21]. The Grading of Recommendation, Assessment, Development and Evaluation (GRADE) approach was used to evaluate the quality of evidence in four domains: strong, moderate, low, and very low. When the two authors (Ziyi Zhao and Leilei Zheng) disagreed, a third investigator (Yun Hu) was consulted for discussion to arrive at a reasonable conclusion.

### Statistical analysis

**Fig. 1:**
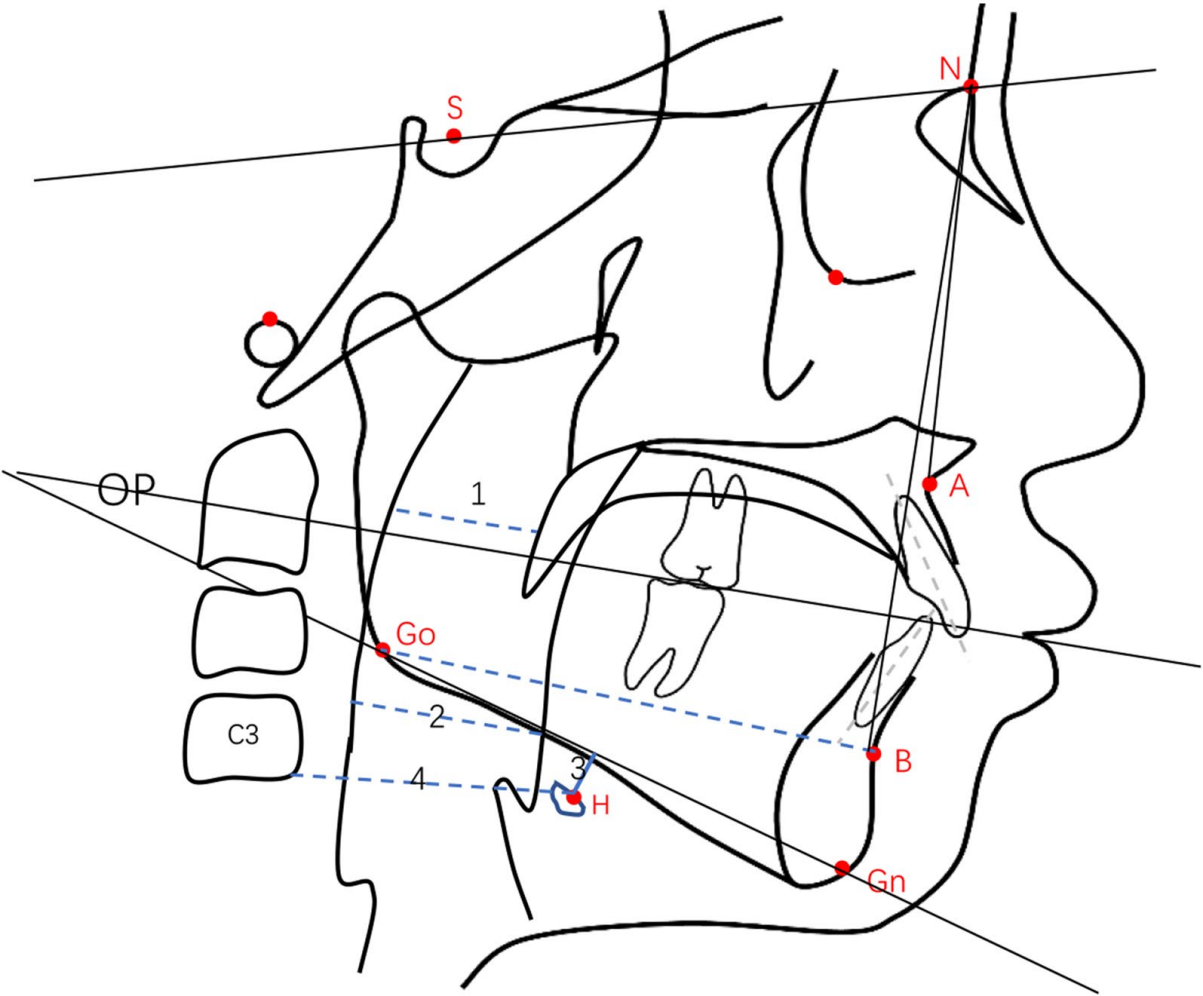
Anatomical drawing showed linear measurements and angles traced for the determination of the cephalometric variables. 1 = SPAS; 2 = PAS; 3 = MPH; 4 = C3-H

Subgroup analysis was performed for all included studies based on the etiology of mouth breathing. The data were analysed using Review Manager 5.3, provided by the Cochrane Collaboration, according to the methods in the Cochrane Handbook for Systematic Reviews of Interventions (version 5.1.0). All the evaluated cephalometric parameters extracted from the included studies were continuous variables. An anatomical drawing was produced and the linear measurements and angles (Fig. 1) were traced out in order to determine the cephalometric variables (Table 1). The mean difference (MDs) with 95% confidence intervals (CIs) were used to construct forest plots for the continuous data. The significance level for the hypothesis test was set at *P* < 0.050. The Cochrane Q test was used to assess the heterogeneity between studies, and Cochrane's test (statistic) was used to evaluate the magnitude of heterogeneity. If heterogeneity was low (*P* > 0.100, I^2^ < 50%), we presented results with fixed-effects model; Otherwise, the random-effects model was adopted for the meta-analysis. If the result was statistically significant (*P* < 0.050) and heterogeneity was high (I^2^ > 75%), sensitivity analyses were conducted by removing each study individually to confirm the effect of the relevant study on the overall mean difference. Funnel plots were used to examine publication bias if the number of included studies exceeded 10.

**Table 1:** Cephalometric variables used in this study

Variable	Description	Diagnostic value
SNA°	Angle formed by the sella-nasion line and line N-point A	Anteroposterior position of the maxilla in relation to the skull base
SNB°	Angle formed by the sella-nasion line and line N-point B	Anteroposterior position of the mandible in relation to the skull base
ANB°	Differences between the SNA and SNB angles	Relation between maxilla and mandible
SN-OP°	Angle formed by the sella-nasion line and the occlusal plane	Inclination of the occlusal plane in relation to the skull base
SN-PP°	Angle formed by the sella-nasion line and palatal plane	The degree of the maxilla inclination in relation to the anterior cranial base
PP-MP°	Angle formed by the palatal plane and mandibular plane	Relates the maxilla to the mandible in the vertical plane
SN-GoGn°	Angle formed by the sella-nasion line and mandibular plane	Inclination of the mandibular plane in relation to the skull base
1.NA°	Angle of inclination of the upper incisor in relation to the NA line	Extent of anterior inclination of the upper incisor
1-NA (mm)	Linear distance between the most salient point of the buccal side of the upper incisor and the NA line measured perpendicularly to the latter	Extent of anterior inclination of the upper incisor
1.NB°	Angle of inclination of the lower incisor in relation to the NB line, which determines the extent of anterior inclination of the lower incisor	Extent of anterior inclination of the lower incisor
1-NB (mm)	Linear distance between the most salient point of the buccal side of the lower incisor and the NB line measured perpendicularly to the latter	Extent of anterior inclination of the lower incisor
Overjet (mm)	Horizontal distance between incisors edges	The degree of overjet
Overbite (mm)	Vertical distance between incisors edges	The degree of overbite
SPAS (mm)	the thickness of the airway behind the soft palate along a line parallel to the Go-B point plane	Obstruction of superior posterior airway space
PAS (mm)	Linear distance between a point at the base of the tongue and another point on the posterior wall of the pharynx, both measured by the extension of a line from point B to point Go	Obstruction of posterior airway space
MP-H (mm)	Linear distance between H, the most anterosuperior point of the hyoid bone, and the mandibular plane measured perpendicularly to the latter	Risk of occlusion, that increases directly with the distance
C3-H(mm)	Linear distance between C3 and H, where C3 is the most anteroinferior point of the third cervical vertebra	Risk of occlusion, that increases inversely with the distance

## Result

### Eligible studies and study characteristics

A total of 1178 records were found by keyword searching in the PubMed (n = 312), Cochrane Library (n = 34), EMBASE (n = 618), Medline (n = 9), Web of Science (n = 200) and Sigle (n = 5) databases. Subsequently, 558 duplicates were removed from the pooled database, and 620 unrelated studies were excluded by screening the titles and abstracts. Following full-text assessments, 22 articles were excluded: 5 articles had no control groups; 5 articles had no cephalometrics; 3 articles had not use the nasal breathing group as control group; 2 studies were case reports; 3 studies did not present metrics of interest; 2 studies were meta-analyses (although we excluded these articles, we included all the original studies); and one study’s subjects were older than the target age range. Finally, a total of 10 studies met the inclusion criteria for meta-analysis [11, 14, 16, 22–28]. Among them, two studies contained subgroups. One article was grouped by sex, and in another study, a second cephalometric analysis was performed a year later in the same population without any intervention. For the latter, we include only the initial measurement data. The publication time of the included studies ranged from 2009 to 2015. The flow diagram of the literature search and review process based on the PRISMA statement is shown in Additional file 3: Appendix B.

### General characteristic of the included studies

In this review, there were a total of 1358 subjects; 643 children with mouth breathing were included in the experimental group and 715 children with normal nasal breathing were included in the control group. The age range included in these studies was 2 to 14 years old. Of the 10 articles included, mouth breathing due to adenoid/tonsil hypertrophy was studied in 6 articles [11, 16, 22, 23, 25, 28], obstructive sleep apnoea syndrome (OSAS) was studied in 2 articles [24, 26], Chronic allergic rhinitis was searched in 1 study [27], and 1 article did not mention the cause [14]. The cephalometric analysis indicators in all the included studies were statistically analysed, and the indicators that appeared 2 times or more were selected for consolidation. The included indicators were SNA, SNB, ANB, SN-OP, SN-PP, PP-MP, Overjet, Overbite, SNGoGn, MP-H, 1-NA, 1. NA, 1. NB, 1-NB, SPAS, PAS, and C3-H. The characteristics of the included studies are summarized in Table 2.

**Table 2:** Characteristics of the included studies

Authors and year	Total	MB	NB	Age (range)	Exposure	Image examination	Type of study	Parameters
Franco, 2015 [22]	226	113	113	3–10	Adenoid/tonsil hypertrophy	Cephalogram	Clinical controlled trial	SNB, ANB, SNGoGn
Mattar, 2011 [16]	73	44	29	3–6	Tonsil hypertrophy	Cephalogram	Clinical controlled trial	SNA, SNB, ANB, SNGoGn,SN-PP,PP-MP
Franco, 2013 [23]	110	55	55	3–10	Adenoid/tonsil hypertrophy	Cephalogram	Clinical controlled trial	SNGoGn
Juliano, 2009 [24]	142	52	90	7–14	OSAS	Cephalogram	Clinical controlled trial	SNA, SNB, ANB, SN-OP, SNGoGn, MP-H, 1-NA, 1.NA, 1.NB, 1-NB, SPAS, PAS, C3-H
Juliano, 2009 [25]	27	15	12	7–14	Adenoid/tonsil hypertrophy	Cephalogram	Clinical controlled trial	SNA, SNB, ANB, SN-OP, SNGoGn, MP-H, 1-NA, 1.NA, 1.NB, 1-NB, SPAS, PAS, C3-H
Juliano, 2013 [26]	144	52	92	7–14	OSAS	Cephalogram	Clinical controlled trial	SNA, SNB, ANB, SN-OP, SNGoGn, MP-H, 1-NA, 1.NA, 1.NB, 1-NB, SPAS, PAS, C3-H
D’Ascanio, 2010 [11]	196	98	98	7–12	Adenoids/tonsil hypertrophy	Cephalogram	Clinical controlled trial	SNA, SNB,SN-PP,PP-MP,Overjet, Overbite
Agostinho, 2015 [27]	70	35	35	5–14	Chronic allergic rhiniti	Cephalogram	Clinical controlled trial	SNA, SNB, ANB, SN-OP, Overjet, Overbite,1-NA, 1.NA, 1.NB, 1-NB
Muñoz, 2014 [14]	118	53	65	6–12	NA	Cephalogram	Clinical controlled trial	SNA, SNB, SNGoGn, SN-OP, 1.NA, 1.NB
Souki, 2012 [28]	252	126	126	2–10	Adenotonsillar hypertrophy	Cephalogram	Clinical controlled trial	SNB, ANB, SNGoGn

*MB* mouth breathing*, NB* nasal breathing, *NA* not available, *OSAS* obstructive sleep apnoea syndrome, *Exposure* the factors of mouth breathing

### Risk of bias assessment

All the included studies were from a specific population, so the representativeness of the included studies was not high. Meanwhile, the included studies were all retrospective studies, so the problem of non-response did not exist. About the bias assessment, four articles had low risk and six articles had medium risk (Table 3). Since there were less than 10 studies included in the meta-analysis, we did not conduct funnel plots or Begg's rank correlation tests.

**Table 3:** Assessment of bias using the risk of bias in non-randomised studies (ROBINS-I) tool

Authors and year	Bias due to confounding	Bias in selection of participants into the study	Bias in classification of interventions	Bias due to deviations from intended interventions	Bias due to missing data	Bias in measurement of outcomes	Bias in selection of the reported result	Overall bias
Franco, 2015 [22]	Moderate	Moderate	Low	Low	Low	Moderate	Low	Moderate
Mattar, 2011 [16]	Low	Low	Low	Low	Low	Low	Low	Low
Franco, 2013 [23]	Moderate	Low	Low	Low	Low	Low	Low	Moderate
Juliano, 2009 [24]	Low	Low	Low	Low	Low	Low	Low	Low
Juliano, 2009 [25]	Moderate	Low	Low	Low	Low	Low	Low	Moderate
Juliano, 2013 [26]	Low	Low	Low	Low	Low	Low	Low	Low
D’Ascanio, 2010 [27]	Low	Low	Low	Low	Low	Moderate	Low	Moderate
Agostinho, 2015 [28]	Low	Moderate	Low	Low	Low	Low	Low	Moderate
Muñoz, 2014 [29]	Low	Moderate	Low	Low	Low	Low	Low	Moderate
Souki, 2012 [30]	Low	Low	Low	Low	Low	Low	Low	Low

### Primary outcome measures

#### Sagittal direction

**Fig. 2:**
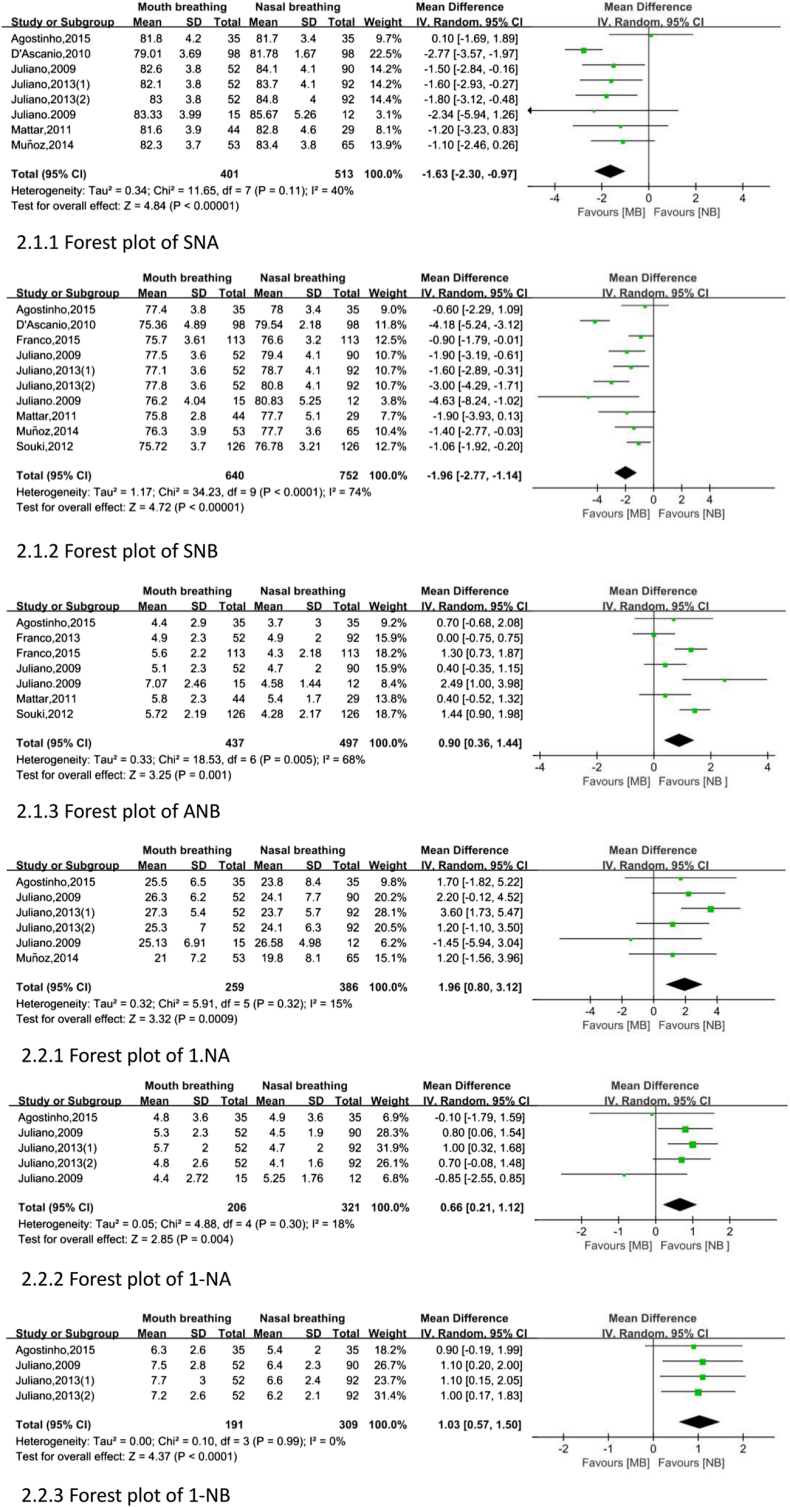
Forest plot of sagittal measurement changes comparing the mouth-breathing groups with the nasal-breathing groups

After the meta-analysis with Review Manager 5.3, 1. NB and Overbite was not statistically significant (fixed: MD, random, 95% CI, *P* > 0.050). As illustrated in Fig. 2, the indicators of sagittal direction are as follows. Two indicators in mouth-breathing children were lower than that in nasal-breathing children: SNA (MD: − 1.63, 95% CI − 2.30 to − 0.97), SNB (MD: − 1.96, 95% CI − 2.77 to − 1.14). However, four parameters showed higher values in children with mouth breathing than in children with nasal breathing: ANB (MD: 0.90, 95% CI 0.36 to 1.44), 1. NA (MD: 1.96, 95% CI 0.80 to 3.12), 1-NA (MD: 0.66, 95% CI 0.21 to 1.12), and 1-NB (MD: 1.03, 95% CI 0.57 to 1.50).

#### Vertical direction

**Fig. 3:**
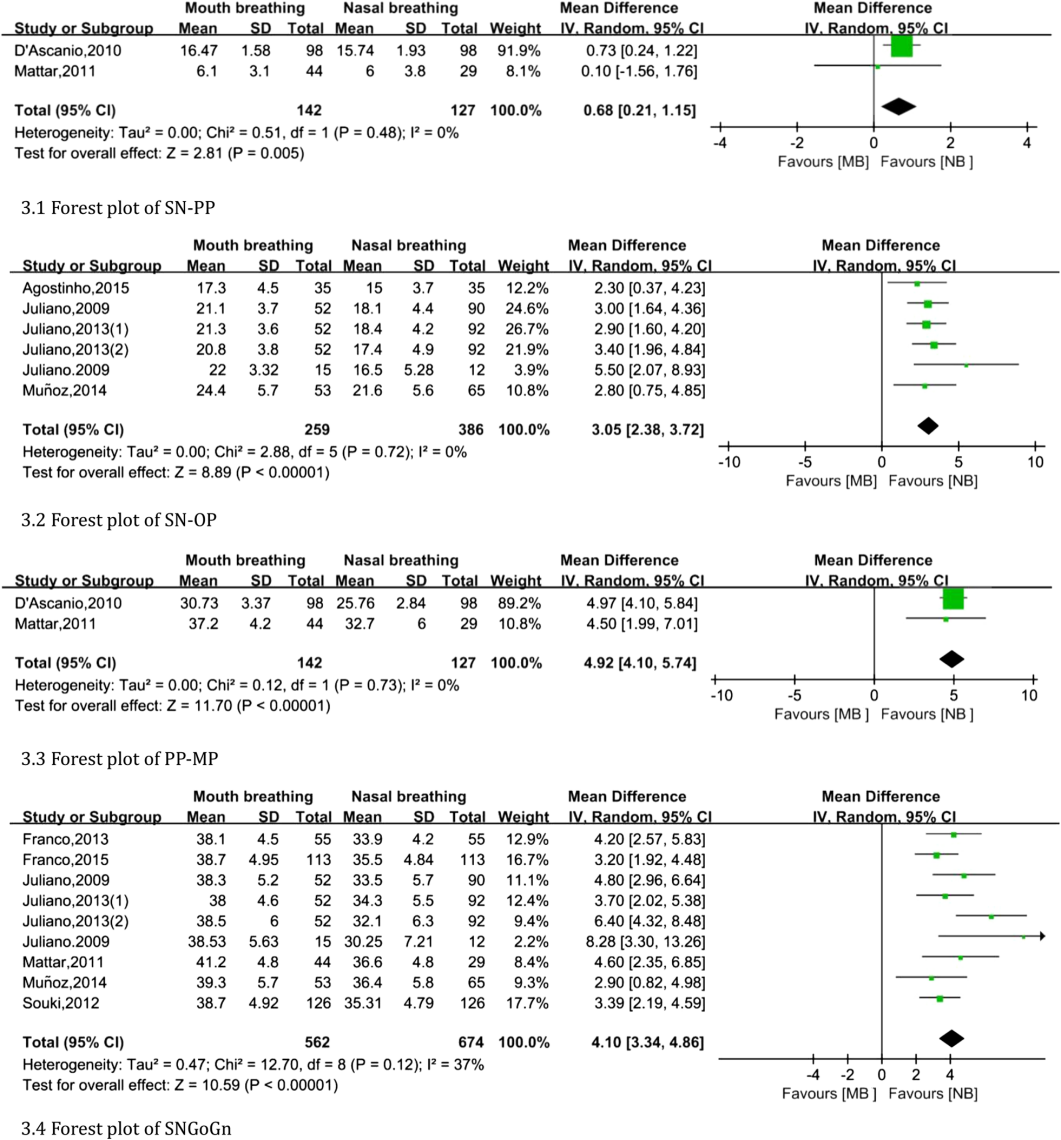
Forest plot of vertical measurement changes comparing the mouth-breathing groups with the nasal-breathing groups

Overjet was not statistically significant (fixed: MD, random, 95% CI, *P* > 0.050). The vertical indicators are shown in Fig. 3. The following indexes were higher in mouth-breathing individuals than in nasal-breathing individuals: SN-PP (MD: 0.68, 95% CI 0.21 to 1.15), SN-OP (MD: 3.05, 95% CI 2.38 to 3.72), PP-MP (MD: 4.92, 95% CI 4.10 to 5.74) and SNGoGn (MD: 4.10, 95% CI 3.34 to 4.86).

#### Airway

**Fig. 4:**
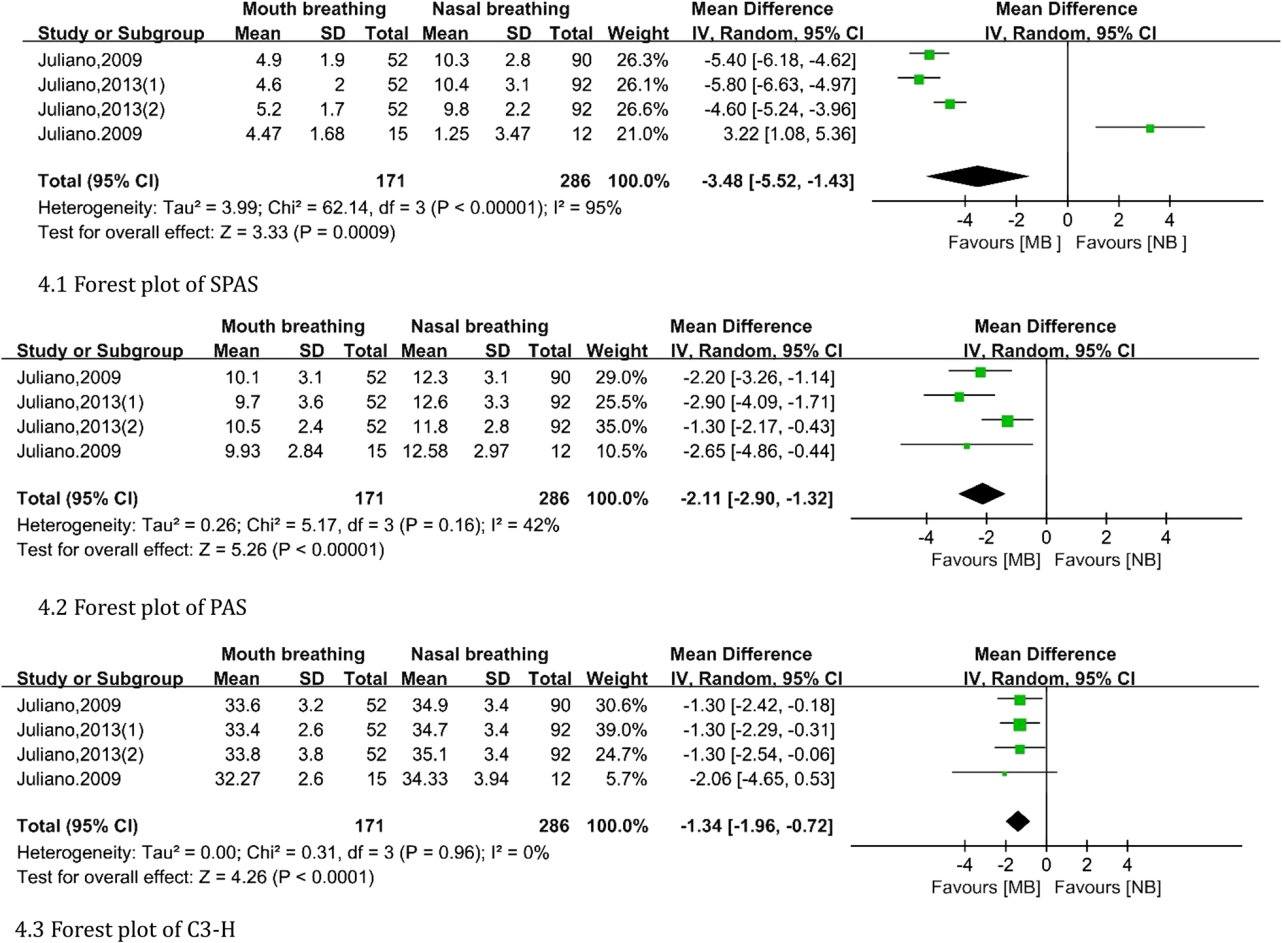
Forest plot of airway changes comparing the mouth-breathing groups with the nasal-breathing groups

After meta-analysis, MP-H were not statistically significant (fixed: MD, random, 95% CI, *P* > 0.050). As shown in Fig. 4, the airway data of children in the mouth breathing group were lower than those in the control group: SPAS (MD: − 3.48, 95% CI − 5.52 to − 1.43), PAS (MD: − 2.11, 95% CI − 2.90 to − 1.32), and C3-H (MD: − 1.34, 95% CI − 1.96 to − 0.72).

The heterogeneity of the other outcomes mentioned above was acceptable.

#### Subgroup analysis

Since there was only one study on mouth breathing caused by allergic rhinitis, so only those studies related to adenoid/tonsil hypertrophy and OSAS were subgroup analyzed. In mouth breathing children with adenoid/tonsil hypertrophy, only ANB, SNB, SN-PP, PP-MP, SNGoGn was statistically significant. As shown in Additional file 4: Appendix C, SNB (MD: − 2.28, 95% CI − 3.81 to − 0.74) is higher than normal children. While, ANB (MD: 1.03, 95% CI 0.35 to 1.71), SN-PP (MD: 0.68, 95% CI 0.21 to 1.15), PP-MP (MD: 4.92, 95% CI 4.10 to 5.74) and SN-GoGn (MD: 3.80, 95% CI 2.94 to 4.65) is lower in mouth breathing children with adenoid/tonsil hypertrophy. As shown in Additional file 5: Appendix D, five outcomes were higher in mouth breathing children with OSAS: SNA (MD: − 1.63, 95% CI − 2.40 to − 0.87), SNB (MD: − 2.17, 95% CI − 3.00 to − 1.33), SPAS (MD: − 5.23, 95% CI − 5.95 to − 4.51), PAS (MD: − 2.06, 95% CI − 2.99 to − 1.14), C3-H (MD: − 1.30, 95% CI − 1.94 to − 0.66). The rest of the results were lower in mouth breathing children with OSAS:SN-OP (MD: 3.08, 95% CI 2.30 to 3.87,), SN-GoGn (MD: 4.87, 95% CI 3.37 to 6.37), 1-NA (MD: 0.85, 95% CI 0.43 to1.27), 1.NA (MD: 2.47, 95% CI 1.05 to 3.89), 1-NB (MD:1.06, 95% CI 0.55 to 1.57). Other indicators were not statistically significant.

## Discussion

### Summary of the result

This systematic review showed that mouth breathing can cause underdevelopment of the jaw in children. From the results, the mandible had obvious rotation tendency according to the position of the skull. The maxilla has the same characteristics as the mandible, which is not consistent with the conclusion of some studies [14, 29]. While, Sousa also pointed out that the maxilla also tends to rotate backward [30]. At the same time, the mandibular plane angle, the palatal plane angle and occlusal plane angle in mouth-breathing children increased, which may adversely affect the temporomandibular joint [31, 32]. Other scholars have also proposed that posterior rotation of the mandible and an increase in the mandibular angle exist in children with mouth breathing, consistent with our results [30, 33–35]. In addition, Kim [33] proposed that children with mouth breathing may also present maxillary shortening. There are also some reports of palatal stenosis in children with nasal obstruction [33, 35, 36]. Mattar proposed that combined with the indexes that were not included, we believed that the ascending ramus of the lower jaw was also underdeveloped in mouth breathing children [16]. The upper anterior teeth showed a tendency for labial inclination. Anterior labial inclination may be caused by an imbalance in the internal and external muscle force due to the lips opening and teeth showing. Although the lower anterior teeth did not have a tendency of labial inclination, the buccal side of the lower incisor is shorter from the lips. From the point of view of the airway, it was obviously narrowed, which may be related to the posterior rotation of the mandible. Posterior inferior rotation of the mandible may compensate for airway stenosis. This conclusion was similar to the results of several studies [30, 34, 35, 37, 38]. Moreover, Juliana's study indicated compensatory growth of the jaw in children with airway obstruction [30]. According to the conclusion of this paper, orthodontists should pay more attention to inducing the normal growth of mandible in children with mouth breathing habits.

### Subgroup analysis

Contrary to the overall conclusion, there was no significant downward rotational trend in the maxilla in mouth breathing children with adenoid/ tonsil hypertrophy. However, in the children with OSAS, the maxillary bone showed a significant tendency of retrodownrotation. In addition, the palatal plane of children with adenoidal hypertrophy developed a posterior downward rotation, which was not present in children with OSAS. This contradiction should be explored by further experiments.

### Summary of the evidence

The GRADE recommendations were moderate for all outcomes except ANB, which is due to risk of bias, plausible counfounding would change the effect (Table 4). There are several confounding factors such as age, sex.

**Table 4:** Summary of findings table according to the GRADE approach

Quality assessment							No of patients		Effect		Quality
No of studies	Design	Risk of bias	Inconsistency	Indirectness	Imprecision	Other considerations	Mouth breathing	Nasal breathing	Relative (95% CI)	Absolute	
*SN-OP (Better indicated by lower values)*											
4	Observational studies	Serious^a^	No serious inconsistency	No serious indirectness	No serious imprecision	Reduced effect for RR > > 1 or RR < < 1^a^	171	286	–	MD 3.2 higher (2.44 to 3.97 higher)	⊕⊕◯◯ Low
*SNA (Better indicated by lower values)*											
5	Observational studies	serious^a^	No serious inconsistency	No serious indirectness	No serious imprecision	Reduced effect for RR > > 1 or RR < < 1^a^	215	315	–	MD 1.61 lower (2.3 to 0.91 lower)	⊕⊕◯◯ Low
*SNB (Better indicated by lower values)*											
6	Observational studies	serious^a^	No serious inconsistency	No serious indirectness	No serious imprecision	Reduced effect for RR > > 1 or RR < < 1^a^	328	428	–	MD 1.92 lower (2.74 to 1.1 lower)	⊕⊕◯◯ Low
*ANB (Better indicated by lower values)*											
5	Observational studies	Serious^a^	No serious inconsistency	No serious indirectness	No serious imprecision	Reduced effect for RR > > 1 or RR < < 1^a^	276	336	–	MD 0.79 higher (0.1 to 1.49 higher)	⊕⊕◯◯ Low
*SNGoGn (Better indicated by lower values)*											
7	Observational studies	Serious^a^	No serious inconsistency	No serious indirectness	No serious imprecision	Reduced effect for RR > > 1 or RR < < 1^a^	383	483	–	MD 4.46 higher (3.52 to 5.39 higher)	⊕⊕◯◯ Low
*1-NA (Better indicated by lower values)*											
4	Observational studies	Serious^a^	No serious inconsistency	No serious indirectness	No serious imprecision	Reduced effect for RR > > 1 or RR < < 1^a^	171	286	–	MD 0.72 higher (0.23 to 1.2 higher)	⊕⊕◯◯ Low
*1.NA (Better indicated by lower values)*											
4	Observational studies	Serious^a^	No serious inconsistency	No serious indirectness	No serious imprecision	Reduced effect for RR > > 1 or RR < < 1^a^	171	286	–	MD 1.98 higher (0.3 to 3.66 higher)	⊕⊕◯◯ low
*1-NB (Better indicated by lower values)*											
3	Observational studies	Serious^a^	No serious inconsistency	No serious indirectness	No serious imprecision	Reduced effect for RR > > 1 or RR < < 1^a^	156	274	-	MD 1.06 higher (0.55 to 1.57 higher)	⊕⊕◯◯ Low
*1.NB (Better indicated by lower values)*											
4	Observational studies	Serious^a^	No serious inconsistency	No serious indirectness	No serious imprecision	Reduced effect for RR > > 1 or RR < < 1^a^	171	286	–	MD 0.41 higher (1.23 lower to 2.04 higher)	⊕⊕◯◯ Low
*SPAS (Better indicated by lower values)*											
3	Observational studies	Serious^a^	No serious inconsistency	No serious indirectness	No serious imprecision	Reduced effect for RR > > 1 or RR < < 1^a^	156	274	–	MD 5.23 lower (5.95 to 4.51 lower)	⊕⊕◯◯ Low
*SN-PP (Better indicated by lower values)*											
2	Observational studies	Serious^a^	No serious inconsistency	No serious indirectness	No serious imprecision	Reduced effect for RR > > 1 or RR < < 1^a^	147	127	–	MD 0.68 higher (0.21 to 1.15 higher)	⊕⊕◯◯ Low
*PAS (Better indicated by lower values)*											
4	Observational studies	Serious^a^	No serious inconsistency	No serious indirectness	No serious imprecision	Reduced effect for RR > > 1 or RR < < 1^a^	171	286	–	MD 2.11 lower (2.9 to 1.32 lower)	⊕⊕◯◯ Low
*PP-MP (Better indicated by lower values)*											
2	Observational studies	No serious risk of bias^a^	No serious inconsistency	No serious indirectness	No serious imprecision	Reduced effect for RR > > 1 or RR < < 1^a^	142	127	–	MD 4.92 higher (4.1 to 5.74 higher)	⊕⊕⊕◯◯ Moderate
*Overjet (Better indicated by lower values)*											
2	Observational studies	Serious^a^	No serious inconsistency	No serious indirectness	No serious imprecision	Reduced effect for RR > > 1 or RR < < 1^a^	133	133	–	MD 0.23 higher (1.39 to 1.84 higher)	⊕⊕◯◯ Low
*C3-H (Better indicated by lower values)*											
4	Observational studies	Serious^a^	No serious inconsistency	No serious indirectness	No serious imprecision	Reduced effect for RR > > 1 or RR < < 1^a^	171	286	–	MD 1.34 lower (1.96 to 0.72 lower)	⊕⊕◯◯ Low
*Overbite (Better indicated by lower values)*											
2	Observational studies	Serious^a^	No serious inconsistency	No serious indirectness	No serious imprecision	Reduced effect for RR > > 1 or RR < < 1 ^a^	133	133	–	MD 1.19 lower (3.24 lower to 0.85 higher)	⊕⊕◯◯ Low
*MP-H (Better indicated by lower values)*											
4	Observational studies	Serious ^a^	No serious inconsistency	No serious indirectness	No serious imprecision	Reduced effect for RR > > 1 or RR < < 1^a^	171	286	–	MD 0.49 higher (0.64 lower to 1.61 higher)	⊕⊕◯◯ Low

Author(s): ziyi zhao, Date: 2020-07-22, Question: Should mouth breathing versus nasal breathing be used for facial growthing?, Settings: Cephalogram, Bibliography: zhao z. Mouth breathing for facial growthing. Cochrane Database of Systematic Reviews [Year], Issue [Issue]

^a^There are several confounding factors such as age, sex, cause of mouth breathing

The relationship between respiratory method and facial skeletal development has long been a topic of interest to paediatricians, otorhinolaryngologists, orthodontists, and other professionals [20, 39–41]. do Nascimento and Becking conducted a systematic review and meta-analysis of the effects of adenoid/tonsil hypertrophy on oral and maxillofacial development before and after oral respiratory surgery [17, 18]. Moreover, Fraga conducted a systematic review and qualitative analysis on the effects of mouth breathing on the occlusal relationship in children [38]. They proposed that before surgery, compared with children with nasal breathing, children with mouth breathing tended to have an increased mandibular plane angle and posterior inferior rotation of the mandible, and most of them had Class II malocclusion. By correcting poor breathing patterns, children's facial development can be improved to a large extent.

According to literature, mouth breathing occurs in 12–55% of children [42–46]. The prevalence of adenoid hypertrophy was 49.70% [47]. The high prevalence of adenoid hypertrophy and mouth breathing reminds us to pay more attention to its prevention. Surgical intervention to remove the cause, and early orthodontic treatment for malocclusion can provide children and adolescents with a higher quality of life. Timely attention to mouth breathing caused by adenoid hypertrophy and other causes can promote the physical and mental health of children.

To the best of our knowledge, this is the first meta-analysis to explore the effects of mouth breathing on facial skeletal development in children. Through a strict and thorough screening process, 10 studies were included. The total sample size of our study was large. The heterogeneity of the results was mostly acceptable. The mouth-breathing group and nasal-breathing group had the same indexes for analysis. Additionally, three authors included all the indicators appearing two times or more in the literature for meta-analysis and reached a conclusion by referring to the indicators not included in the analysis to ensure the reliability of the conclusions. Altogether, the results of this meta-analysis are credible.

Nevertheless, certain limitations exist. Considering that children's facial skeletal development is closely related to age and sex, heterogeneity may be derived from the age and sex of the research subjects. We tried to conduct subgroup analyses considering age and sex but found that the included literature in this study included overlapping ages and did not stratify data by sex, so this condition was not met (Forest plot for children aged 2–10 and 7–14 are shown in Additional file 6: Appendix E1 and Additional file 7: Appendix E2). Although there was an age range in the included literature, only a few studies conducted cephalometric analyses by age group, so subgroup analysis was not feasible in this meta-analysis. Therefore, the effects of facial skeletal development at various stages of growth and development could not be determined. Additionally, the growth and development peaks of the sexes differ. Considering the low heterogeneity of the included indicators in this paper, it was confirmed that age and gender had little influence on this study. So, the data are still reliable. Nevertheless, we are willing to conduct a long-term literature review and relevant clinical studies to explore this issue.

## Conclusion

The results showed that the mandible and maxilla rotated backward and downward, and the occlusal plane was steep in mouth breathing children. In addition, mouth breathing presented a tendency of labial inclination of the upper anterior teeth. Airway stenosis was common in mouth-breathing children. Contrary to children with OSAS, there was no significant downward rotational trend in the maxilla in mouth breathing children with adenoid/ tonsil hypertrophy. At the same time, the palatal plane of children with adenoidal hypertrophy developed a posterior downward rotation, which was not present in children with OSAS.

## Supplementary Information

**Additional file 1:** The PRISMA checklist.

**Additional file 2:** Electronic search strategy for PubMed.

**Additional file 3:** Study selection flow diagram (PRISMA).

**Additional file 4:** Forest plot of mouth breathing caused by adenoid/tonsil hypertrophy.

**Additional file 5:** Forest plot of mouth breathing caused by OSAS.

**Additional file 6:** Forest plot  for children aged 2–10.

**Additional file 7:** Forest plot for children aged 7–14.

## Data Availability

All data generated or analysed during this study are included in this published article and its supplementary information files.
