# Supplementary material for: Effects of mouth breathing on facial skeletal development in children: a systematic review and meta-analysis
Source: BMC Oral Health. 2021 Mar 10;21:108. doi: 10.1186/s12903-021-01458-7 (PMC7944632; doi:10.1186/s12903-021-01458-7)
Supplement: Supplementary file 2 — Additional file 2: Electronic search strategy for PubMed. [file 12903_2021_1458_MOESM2_ESM.docx]

Search **(((((((((((((((((((((Malocclusions[Title/Abstract]) OR Tooth Crowding[Title/Abstract]) OR Crowding, Tooth[Title/Abstract]) OR Crowdings, Tooth[Title/Abstract]) OR Crossbite[Title/Abstract]) OR Crossbites[Title/Abstract]) OR Cross Bite[Title/Abstract]) OR Bite, Cross[Title/Abstract]) OR Bites, Cross[Title/Abstract]) OR Cross Bites[Title/Abstract]) OR Angle's Classification[Title/Abstract]) OR Angle Classification[Title/Abstract]) OR Angles Classification[Title/Abstract]) OR Classification, Angle's[Title/Abstract]) OR Dentofacial growth[Title/Abstract]) OR Facial growth[Title/Abstract])) OR "Malocclusion"[Mesh]) OR (((((((((((Mandibles[Title/Abstract]) OR Mylohyoid Ridge[Title/Abstract]) OR Mylohyoid Ridges[Title/Abstract]) OR Ridge, Mylohyoid[Title/Abstract]) OR Ridges, Mylohyoid[Title/Abstract]) OR Mylohyoid Groove[Title/Abstract]) OR Groove, Mylohyoid[Title/Abstract]) OR Grooves, Mylohyoid[Title/Abstract]) OR Mylohyoid Grooves[Title/Abstract])) OR "Mandible"[Mesh])) OR ((((((((Maxillas[Title/Abstract]) OR Maxillary Bone[Title/Abstract]) OR Bone, Maxillary[Title/Abstract]) OR Bones, Maxillary[Title/Abstract]) OR Maxillary Bones[Title/Abstract]) AND Maxillae[Title/Abstract])) OR "Maxilla"[Mesh]))) AND (("Mouth Breathing"[Mesh]) OR (((Breathing, Mouth[Title/Abstract]) OR Breathings, Mouth[Title/Abstract]) OR Mouth Breathings[Title/Abstract]))**
