# Supplementary figures and images for: Effects of mouth breathing on facial skeletal development in children: a systematic review and meta-analysis
Source: BMC Oral Health. 2021 Mar 10;21:108. doi: 10.1186/s12903-021-01458-7 (PMC7944632; doi:10.1186/s12903-021-01458-7)

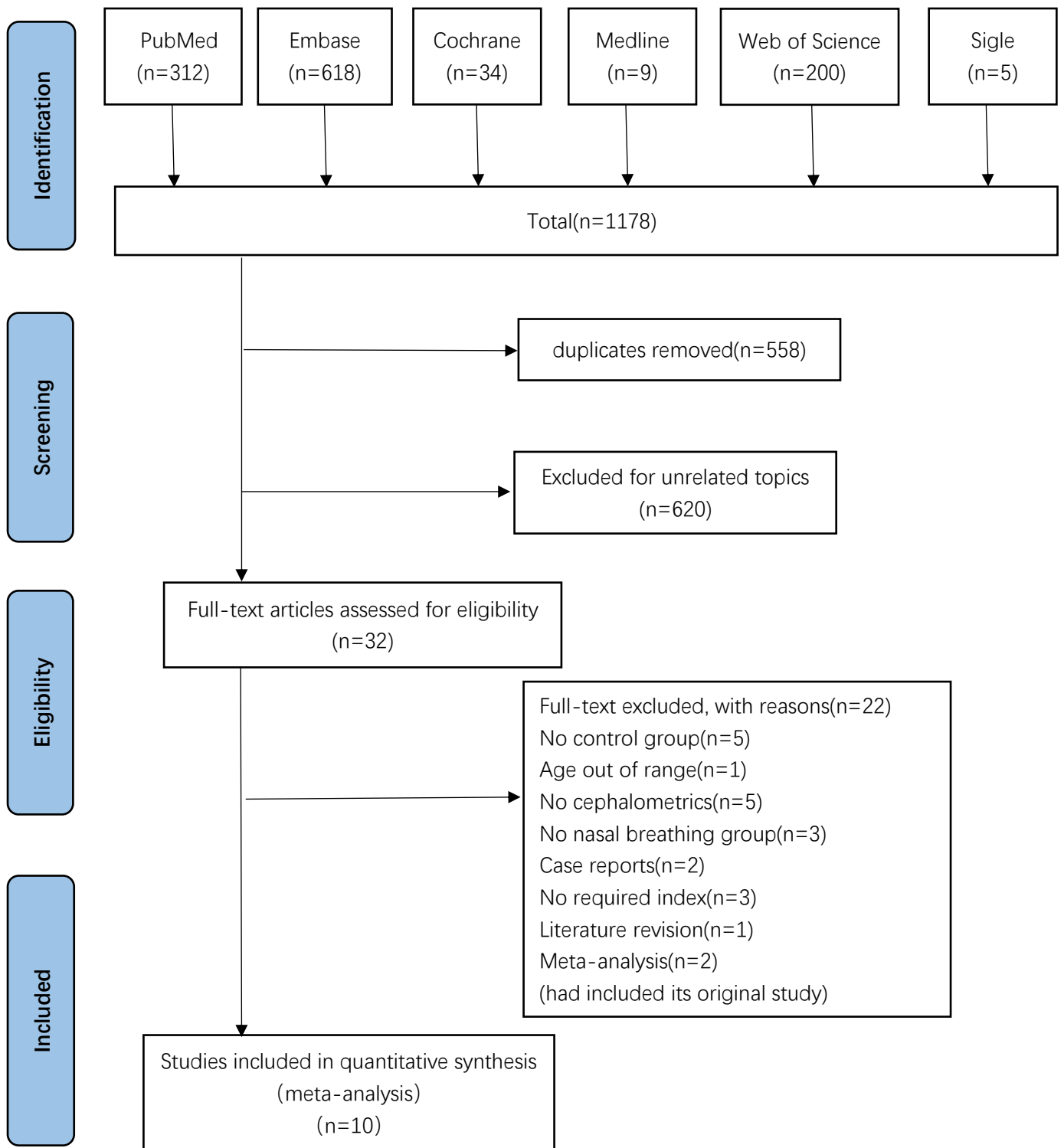

Supplement: Supplementary file 3 — Additional file 3: Study selection flow diagram (PRISMA). [file 12903_2021_1458_MOESM3_ESM.pdf]

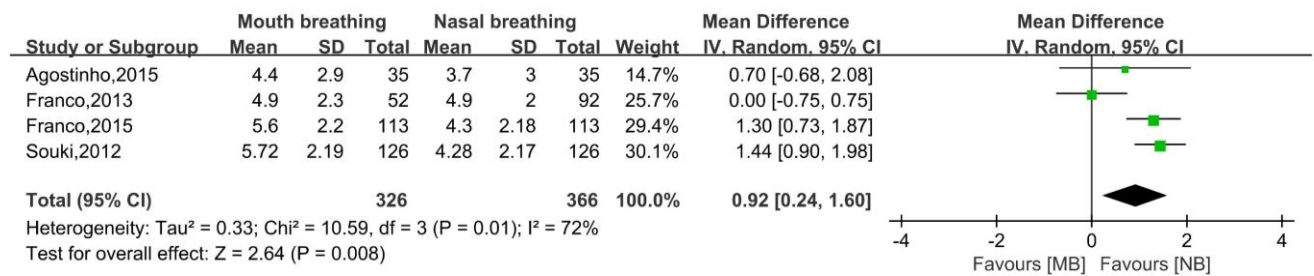

E1.1 Forest plot of ANB

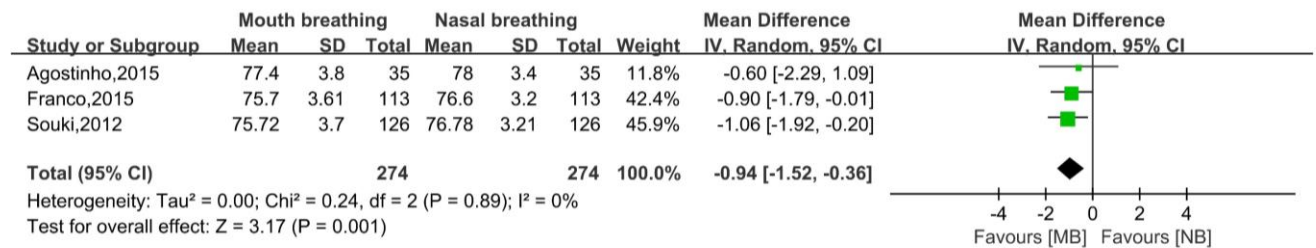

E1.2 Forest plot of SNB

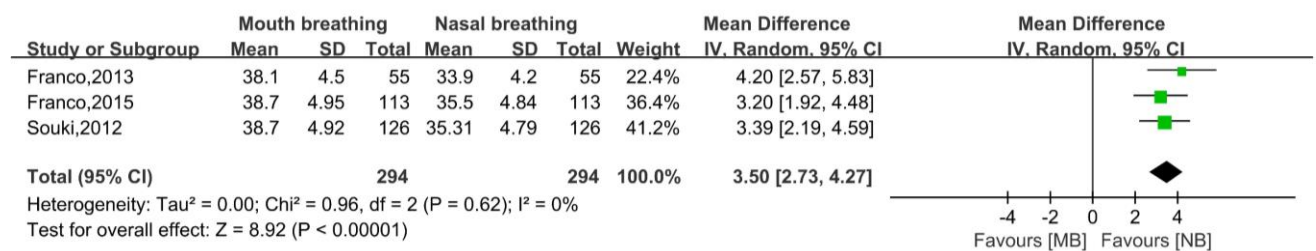

E1.3 Forest plot of SN-GoGn

Supplement: Supplementary file 6 — Additional file 6: Forest plot for children aged 2–10. [file 12903_2021_1458_MOESM6_ESM.pdf]
