# Supplementary material for: Effects of mouth breathing on facial skeletal development in children: a systematic review and meta-analysis
Source: BMC Oral Health. 2021 Mar 10;21:108. doi: 10.1186/s12903-021-01458-7 (PMC7944632; doi:10.1186/s12903-021-01458-7)
Supplement: Supplementary file 4 — Additional file 4: Forest plot of mouth breathing caused by adenoid/tonsil hypertrophy. [file 12903_2021_1458_MOESM4_ESM.pdf]

Study or Subgroup	Mouth breathing Mean	SD	Total	Nasal breathing Mean	SD	Total	Weight	Mean Difference IV, Random, 95% CI
Franco,2013	4.9	2.3	52	4.9	2	92	21.2%	0.00 [-0.75, 0.75]
Franco,2015	5.6	2.2	113	4.3	2.18	113	23.8%	1.30 [0.73, 1.87]
Juliano.2009	7.07	2.46	15	4.58	1.44	12	12.1%	2.49 [1.00, 3.98]
Mattar,2011	5.8	2.3	44	5.4	1.7	29	18.8%	0.40 [-0.52, 1.32]
Souki,2012	5.72	2.19	126	4.28	2.17	126	24.2%	1.44 [0.90, 1.98]
**Total (95% CI)**			**350**			**372**	**100.0%**	**1.03 [0.35, 1.71]**

Heterogeneity: Tau² = 0.42; Chi² = 15.86, df = 4 (P = 0.003); I² = 75%
Test for overall effect: Z = 2.98 (P = 0.003)

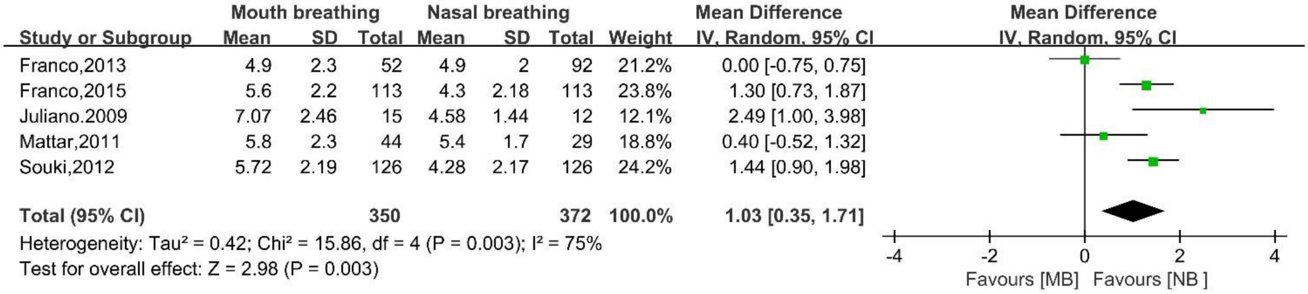

C.1 Forest plot of ANB

Study or Subgroup	Mouth breathing Mean	SD	Total	Nasal breathing Mean	SD	Total	Weight	Mean Difference IV, Random, 95% CI
D'Ascanio,2010	75.36	4.89	98	79.54	2.18	98	23.2%	-4.18 [-5.24, -3.12]
Franco,2015	75.7	3.61	113	76.6	3.2	113	24.0%	-0.90 [-1.79, -0.01]
Juliano.2009	76.2	4.04	15	80.83	5.25	12	10.7%	-4.63 [-8.24, -1.02]
Mattar,2011	75.8	2.8	44	77.7	5.1	29	17.9%	-1.90 [-3.93, 0.13]
Souki,2012	75.72	3.7	126	76.78	3.21	126	24.2%	-1.06 [-1.92, -0.20]
**Total (95% CI)**			**396**			**378**	**100.0%**	**-2.28 [-3.81, -0.74]**

Heterogeneity: Tau² = 2.35; Chi² = 28.51, df = 4 (P < 0.00001); I² = 86%
Test for overall effect: Z = 2.91 (P = 0.004)

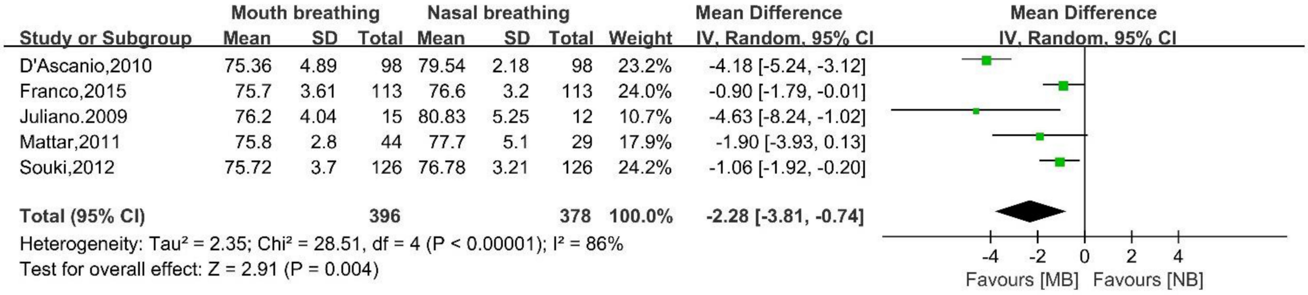

C.2 Forest plot of SNB

Study or Subgroup	Mouth breathing Mean	SD	Total	Nasal breathing Mean	SD	Total	Weight	Mean Difference IV, Random, 95% CI
D'Ascanio,2010	16.47	1.58	98	15.74	1.93	98	91.9%	0.73 [0.24, 1.22]
Mattar,2011	6.1	3.1	44	6	3.8	29	8.1%	0.10 [-1.56, 1.76]
**Total (95% CI)**			**142**			**127**	**100.0%**	**0.68 [0.21, 1.15]**

Heterogeneity: Tau² = 0.00; Chi² = 0.51, df = 1 (P = 0.48); I² = 0%
Test for overall effect: Z = 2.81 (P = 0.005)

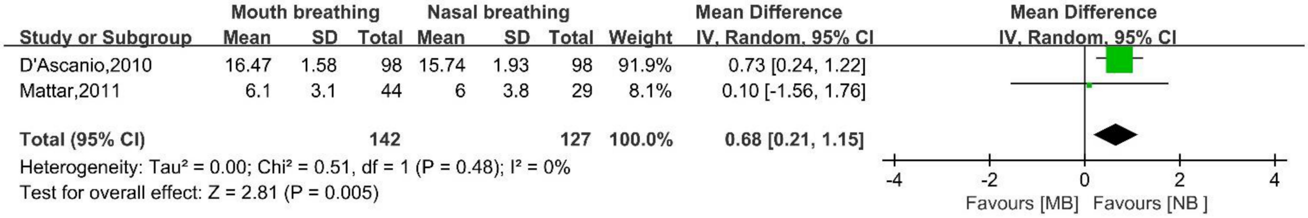

C.3 Forest plot of SN-PP

Study or Subgroup	Mouth breathing Mean	SD	Total	Nasal breathing Mean	SD	Total	Weight	Mean Difference IV, Random, 95% CI
D'Ascanio,2010	30.73	3.37	98	25.76	2.84	98	89.2%	4.97 [4.10, 5.84]
Mattar,2011	37.2	4.2	44	32.7	6	29	10.8%	4.50 [1.99, 7.01]
**Total (95% CI)**			**142**			**127**	**100.0%**	**4.92 [4.10, 5.74]**

Heterogeneity: Tau² = 0.00; Chi² = 0.12, df = 1 (P = 0.73); I² = 0%
Test for overall effect: Z = 11.70 (P < 0.00001)

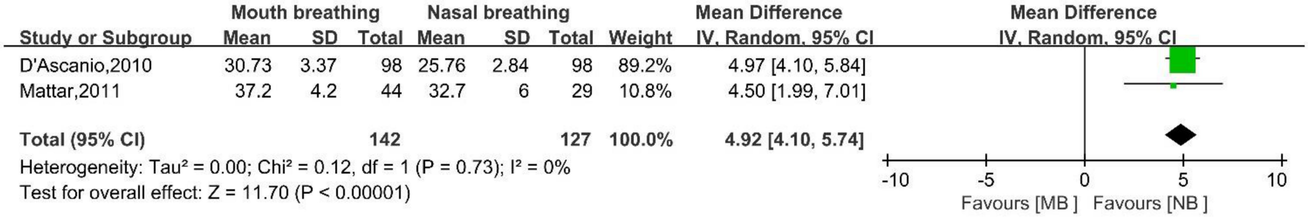

C.4 Forest plot of PP-MP

Study or Subgroup	Mouth breathing Mean	SD	Total	Nasal breathing Mean	SD	Total	Weight	Mean Difference IV, Random, 95% CI
Franco,2013	38.1	4.5	55	33.9	4.2	55	21.3%	4.20 [2.57, 5.83]
Franco,2015	38.7	4.95	113	35.5	4.84	113	30.3%	3.20 [1.92, 4.48]
Juliano.2009	38.53	5.63	15	30.25	7.21	12	2.9%	8.28 [3.30, 13.26]
Mattar,2011	41.2	4.8	44	36.6	4.8	29	12.5%	4.60 [2.35, 6.85]
Souki,2012	38.7	4.92	126	35.31	4.79	126	33.0%	3.39 [2.19, 4.59]
**Total (95% CI)**			**353**			**335**	**100.0%**	**3.80 [2.94, 4.65]**

Heterogeneity: Tau² = 0.20; Chi² = 5.08, df = 4 (P = 0.28); I² = 21%
Test for overall effect: Z = 8.71 (P < 0.00001)

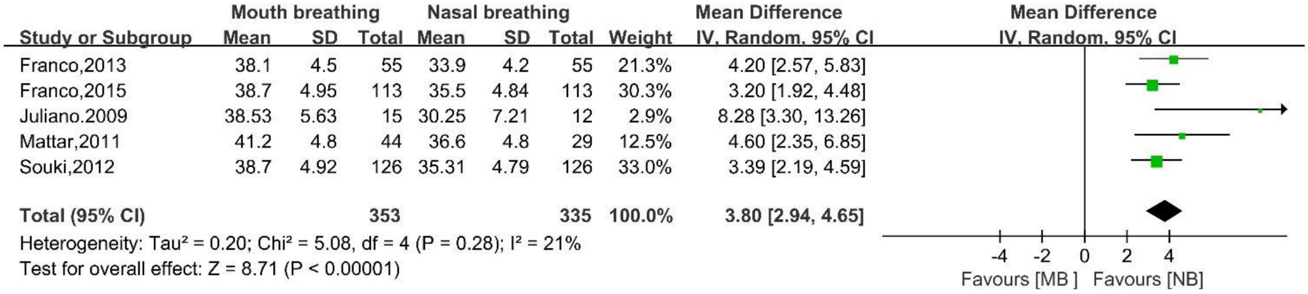

C.5 Forest plot of SNGoGn
