# Supplementary material for: Effects of mouth breathing on facial skeletal development in children: a systematic review and meta-analysis
Source: BMC Oral Health. 2021 Mar 10;21:108. doi: 10.1186/s12903-021-01458-7 (PMC7944632; doi:10.1186/s12903-021-01458-7)
Supplement: Supplementary file 5 — Additional file 5: Forest plot of mouth breathing caused by OSAS. [file 12903_2021_1458_MOESM5_ESM.pdf]

	Mouth breathing			Nasal breathing				Mean Difference
Study or Subgroup	Mean	SD	Total	Mean	SD	Total	Weight	IV, Random, 95% CI
Juliano,2009	82.6	3.8	52	84.1	4.1	90	32.9%	-1.50 [-2.84, -0.16]
Juliano,2013(1)	82.1	3.8	52	83.7	4.1	92	33.2%	-1.60 [-2.93, -0.27]
Juliano,2013(2)	83	3.8	52	84.8	4	92	33.9%	-1.80 [-3.12, -0.48]
**Total (95% CI)**			**156**			**274**	**100.0%**	**-1.63 [-2.40, -0.87]**

Heterogeneity: Tau² = 0.00; Chi² = 0.10, df = 2 (P = 0.95); I² = 0%
Test for overall effect: Z = 4.18 (P < 0.0001)

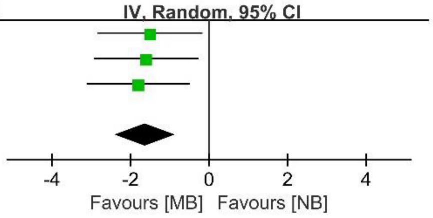

D.1 Forest plot of SNA

	Mouth breathing			Nasal breathing				Mean Difference
Study or Subgroup	Mean	SD	Total	Mean	SD	Total	Weight	IV, Random, 95% CI
Juliano,2009	77.5	3.6	52	79.4	4.1	90	33.2%	-1.90 [-3.19, -0.61]
Juliano,2013(1)	77.1	3.6	52	78.7	4.1	92	33.4%	-1.60 [-2.89, -0.31]
Juliano,2013(2)	77.8	3.6	52	80.8	4.1	92	33.4%	-3.00 [-4.29, -1.71]
**Total (95% CI)**			**156**			**274**	**100.0%**	**-2.17 [-3.00, -1.33]**

Heterogeneity: Tau² = 0.11; Chi² = 2.51, df = 2 (P = 0.28); I² = 20%
Test for overall effect: Z = 5.09 (P < 0.00001)

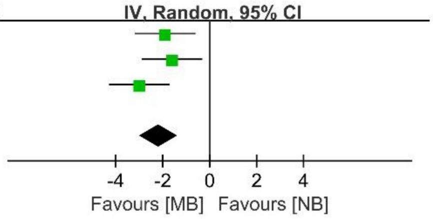

D.2 Forest plot of SNB

	Mouth breathing			Nasal breathing				Mean Difference
Study or Subgroup	Mean	SD	Total	Mean	SD	Total	Weight	IV, Random, 95% CI
Juliano,2009	21.1	3.7	52	18.1	4.4	90	33.6%	3.00 [1.64, 4.36]
Juliano,2013(1)	21.3	3.6	52	18.4	4.2	92	36.5%	2.90 [1.60, 4.20]
Juliano,2013(2)	20.8	3.8	52	17.4	4.9	92	29.9%	3.40 [1.96, 4.84]
**Total (95% CI)**			**156**			**274**	**100.0%**	**3.08 [2.30, 3.87]**

Heterogeneity: Tau² = 0.00; Chi² = 0.28, df = 2 (P = 0.87); I² = 0%
Test for overall effect: Z = 7.69 (P < 0.00001)

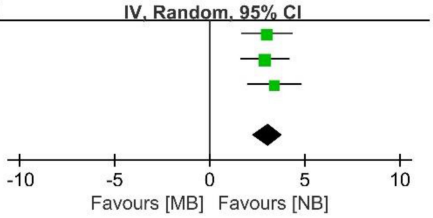

D.3 Forest plot of SN-OP

	Mouth breathing			Nasal breathing				Mean Difference
Study or Subgroup	Mean	SD	Total	Mean	SD	Total	Weight	IV, Random, 95% CI
Juliano,2009	38.3	5.2	52	33.5	5.7	90	33.7%	4.80 [2.96, 6.64]
Juliano,2013(1)	38	4.6	52	34.3	5.5	92	36.7%	3.70 [2.02, 5.38]
Juliano,2013(2)	38.5	6	52	32.1	6.3	92	29.6%	6.40 [4.32, 8.48]
**Total (95% CI)**			**156**			**274**	**100.0%**	**4.87 [3.37, 6.37]**

Heterogeneity: Tau² = 0.86; Chi² = 3.92, df = 2 (P = 0.14); I² = 49%
Test for overall effect: Z = 6.35 (P < 0.00001)

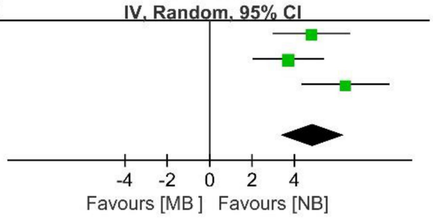

D.4 Forest plot of SNGoGN

	Mouth breathing			Nasal breathing				Mean Difference
Study or Subgroup	Mean	SD	Total	Mean	SD	Total	Weight	IV, Random, 95% CI
Juliano,2009	26.3	6.2	52	24.1	7.7	90	29.5%	2.20 [-0.12, 4.52]
Juliano,2013(1)	27.3	5.4	52	23.7	5.7	92	40.6%	3.60 [1.73, 5.47]
Juliano,2013(2)	25.3	7	52	24.1	6.3	92	29.9%	1.20 [-1.10, 3.50]
**Total (95% CI)**			**156**			**274**	**100.0%**	**2.47 [1.05, 3.89]**

Heterogeneity: Tau² = 0.37; Chi² = 2.62, df = 2 (P = 0.27); I² = 24%
Test for overall effect: Z = 3.42 (P = 0.0006)

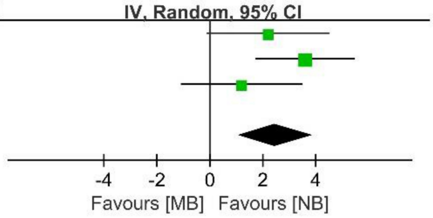

D.5 Forest plot of 1.NA

	MB			NB				Mean Difference
Study or Subgroup	Mean	SD	Total	Mean	SD	Total	Weight	IV, Random, 95% CI
Juliano,2009	31.8	5.8	52	30.9	5.4	90	32.7%	0.90 [-1.03, 2.83]
Juliano,2013(1)	33	6.1	52	30.9	5.7	92	31.3%	2.10 [0.07, 4.13]
Juliano,2013(2)	30.4	5.1	52	31.1	5.1	92	36.0%	-0.70 [-2.43, 1.03]
**Total (95% CI)**			**156**			**274**	**100.0%**	**0.70 [-0.91, 2.31]**

Heterogeneity: Tau² = 1.10; Chi² = 4.36, df = 2 (P = 0.11); I² = 54%
Test for overall effect: Z = 0.85 (P = 0.40)

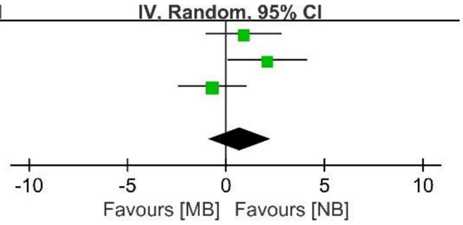

D.6 Forest plot of 1.NB

Study or Subgroup	Mouth breathing Mean	SD	Total	Nasal breathing Mean	SD	Total	Weight	Mean Difference IV, Random, 95% CI
Juliano,2009	5.3	2.3	52	4.5	1.9	90	32.5%	0.80 [0.06, 1.54]
Juliano,2013(1)	5.7	2	52	4.7	2	92	38.3%	1.00 [0.32, 1.68]
Juliano,2013(2)	4.8	2.6	52	4.1	1.6	92	29.2%	0.70 [-0.08, 1.48]
**Total (95% CI)**			**156**			**274**	**100.0%**	**0.85 [0.43, 1.27]**

Heterogeneity: Tau² = 0.00; Chi² = 0.35, df = 2 (P = 0.84); I² = 0%
Test for overall effect: Z = 3.95 (P < 0.0001)

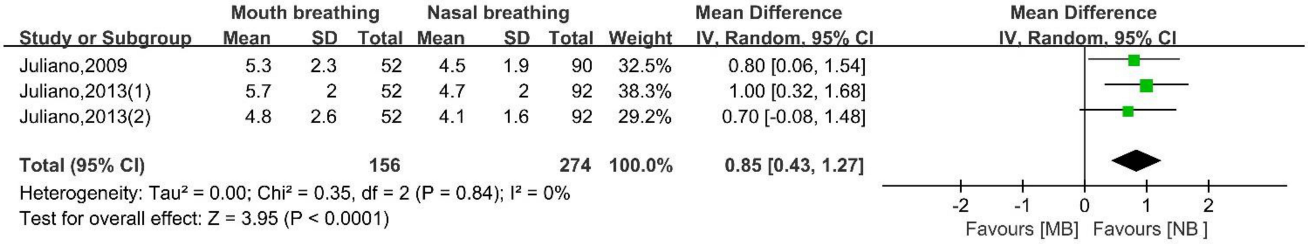

D.7 Forest plot of 1-NA

Study or Subgroup	Mouth breathing Mean	SD	Total	Nasal breathing Mean	SD	Total	Weight	Mean Difference IV, Random, 95% CI
Juliano,2009	7.5	2.8	52	6.4	2.3	90	32.6%	1.10 [0.20, 2.00]
Juliano,2013(1)	7.7	3	52	6.6	2.4	92	29.0%	1.10 [0.15, 2.05]
Juliano,2013(2)	7.2	2.6	52	6.2	2.1	92	38.4%	1.00 [0.17, 1.83]
**Total (95% CI)**			**156**			**274**	**100.0%**	**1.06 [0.55, 1.57]**

Heterogeneity: Tau² = 0.00; Chi² = 0.03, df = 2 (P = 0.98); I² = 0%
Test for overall effect: Z = 4.06 (P < 0.0001)

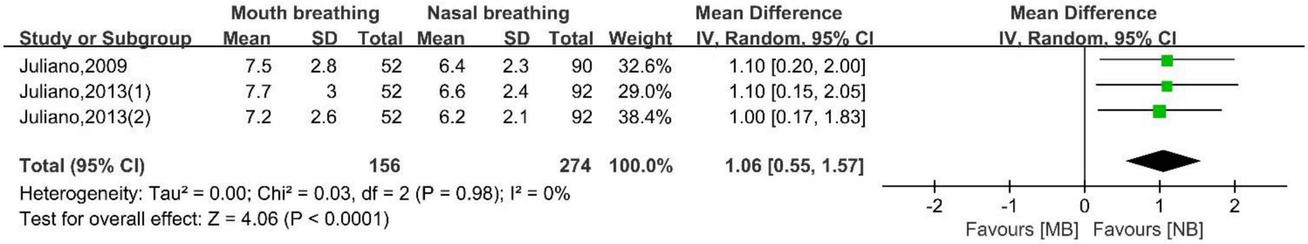

D.8 Forest plot of 1-NB

Study or Subgroup	Mouth breathing Mean	SD	Total	Nasal breathing Mean	SD	Total	Weight	Mean Difference IV, Random, 95% CI
Juliano,2009	4.9	1.9	52	10.3	2.8	90	32.5%	-5.40 [-6.18, -4.62]
Juliano,2013(1)	4.6	2	52	10.4	3.1	92	30.7%	-5.80 [-6.63, -4.97]
Juliano,2013(2)	5.2	1.7	52	9.8	2.2	92	36.8%	-4.60 [-5.24, -3.96]
**Total (95% CI)**			**156**			**274**	**100.0%**	**-5.23 [-5.95, -4.51]**

Heterogeneity: Tau² = 0.26; Chi² = 5.52, df = 2 (P = 0.06); I² = 64%
Test for overall effect: Z = 14.27 (P < 0.00001)

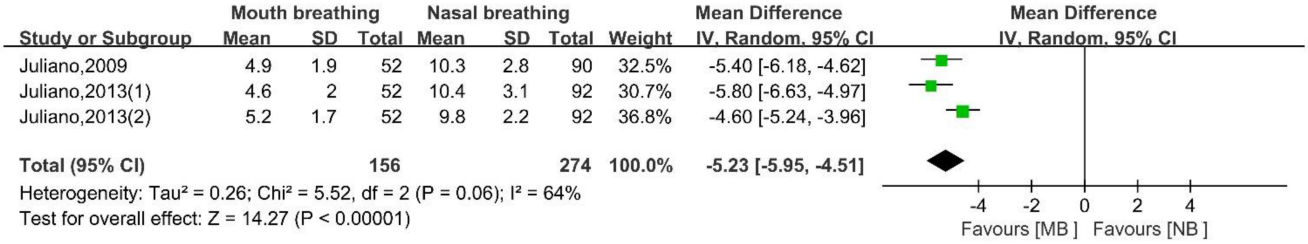

D.9 Forest plot of SPAS

Study or Subgroup	Mouth breathing Mean	SD	Total	Nasal breathing Mean	SD	Total	Weight	Mean Difference IV, Random, 95% CI
Juliano,2009	10.1	3.1	52	12.3	3.1	90	32.7%	-2.20 [-3.26, -1.14]
Juliano,2013(1)	9.7	3.6	52	12.6	3.3	92	29.4%	-2.90 [-4.09, -1.71]
Juliano,2013(2)	10.5	2.4	52	11.8	2.8	92	38.0%	-1.30 [-2.17, -0.43]
**Total (95% CI)**			**156**			**274**	**100.0%**	**-2.06 [-2.99, -1.14]**

Heterogeneity: Tau² = 0.39; Chi² = 4.82, df = 2 (P = 0.09); I² = 59%
Test for overall effect: Z = 4.37 (P < 0.0001)

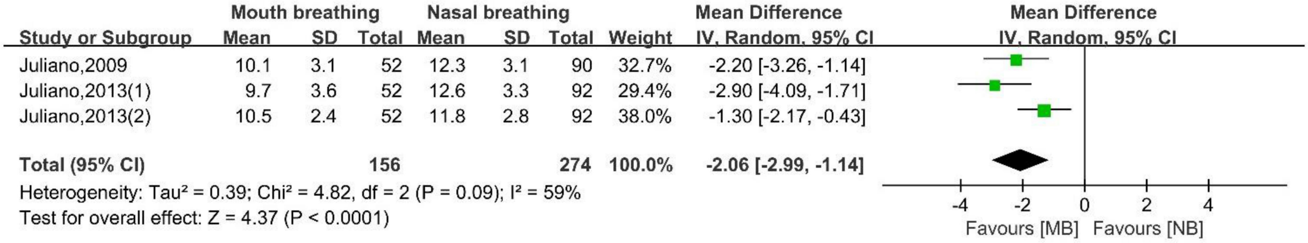

D.10 Forest plot of PAS

Study or Subgroup	Mouth breathing Mean	SD	Total	Nasal breathing Mean	SD	Total	Weight	Mean Difference IV, Random, 95% CI
Juliano,2009	33.6	3.2	52	34.9	3.4	90	32.5%	-1.30 [-2.42, -0.18]
Juliano,2013(1)	33.4	2.6	52	34.7	3.4	92	41.3%	-1.30 [-2.29, -0.31]
Juliano,2013(2)	33.8	3.8	52	35.1	3.4	92	26.2%	-1.30 [-2.54, -0.06]
**Total (95% CI)**			**156**			**274**	**100.0%**	**-1.30 [-1.94, -0.66]**

Heterogeneity: Tau² = 0.00; Chi² = 0.00, df = 2 (P = 1.00); I² = 0%
Test for overall effect: Z = 4.00 (P < 0.0001)

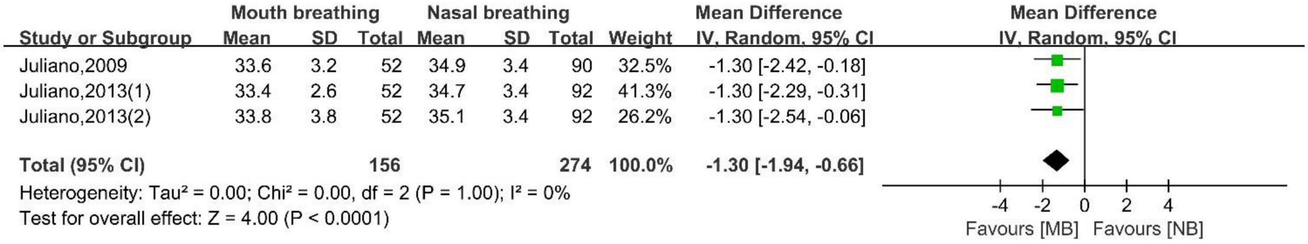

D.11 Forest plot of C3-H
