# Supplementary material for: Effects of mouth breathing on facial skeletal development in children: a systematic review and meta-analysis
Source: BMC Oral Health. 2021 Mar 10;21:108. doi: 10.1186/s12903-021-01458-7 (PMC7944632; doi:10.1186/s12903-021-01458-7)
Supplement: Supplementary file 7 — Additional file 7: Forest plot for children aged 7–14. [file 12903_2021_1458_MOESM7_ESM.pdf]

Study or Subgroup	Mouth breathing Mean	SD	Total	Nasal breathing Mean	SD	Total	Weight	Mean Difference IV, Random, 95% CI
Juliano,2009	5.1	2.3	52	4.7	2	90	39.4%	0.40 [-0.35, 1.15]
Juliano.2009	7.07	2.46	15	4.58	1.44	12	24.9%	2.49 [1.00, 3.98]
Mattar,2011	5.8	2.3	44	5.4	1.7	29	35.8%	0.40 [-0.52, 1.32]
**Total (95% CI)**			**111**			**131**	**100.0%**	**0.92 [-0.14, 1.97]**

Heterogeneity: Tau² = 0.59; Chi² = 6.58, df = 2 (P = 0.04); I² = 70%
Test for overall effect: Z = 1.71 (P = 0.09)

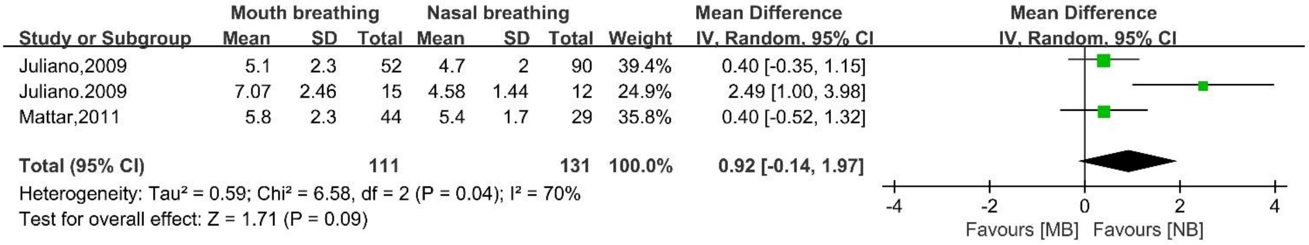

E2.1 Forest plot of ANB

Study or Subgroup	Mouth breathing Mean	SD	Total	Nasal breathing Mean	SD	Total	Weight	Mean Difference IV, Random, 95% CI
D'Ascanio,2010	75.36	4.89	98	79.54	2.18	98	21.8%	-4.18 [-5.24, -3.12]
Juliano,2009	77.5	3.6	52	79.4	4.1	90	19.5%	-1.90 [-3.19, -0.61]
Juliano,2013(1)	77.1	3.6	52	78.7	4.1	92	19.6%	-1.60 [-2.89, -0.31]
Juliano,2013(2)	77.8	3.6	52	80.8	4.1	92	19.6%	-3.00 [-4.29, -1.71]
Juliano.2009	76.2	4.04	15	80.83	5.25	12	6.2%	-4.63 [-8.24, -1.02]
Mattar,2011	75.8	2.8	44	77.7	5.1	29	13.3%	-1.90 [-3.93, 0.13]
**Total (95% CI)**			**313**			**413**	**100.0%**	**-2.72 [-3.74, -1.71]**

Heterogeneity: Tau² = 0.94; Chi² = 13.54, df = 5 (P = 0.02); I² = 63%
Test for overall effect: Z = 5.26 (P < 0.00001)

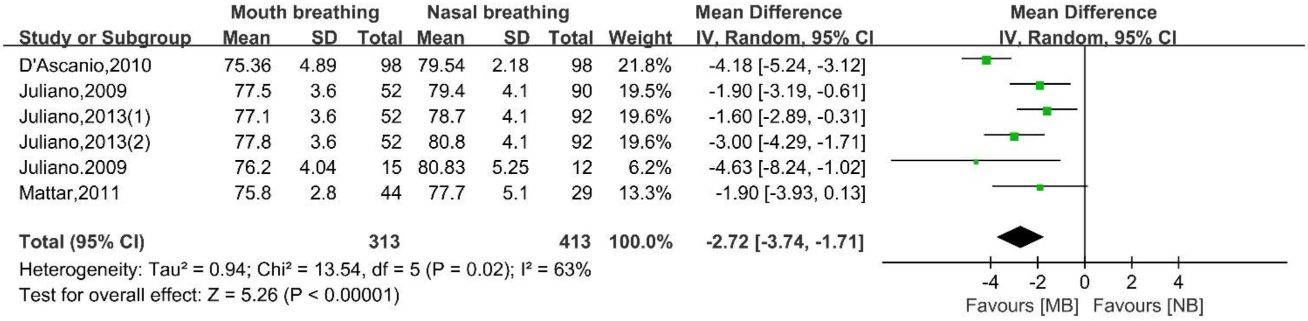

E2.2 Forest plot of SNB

Study or Subgroup	Mouth breathing Mean	SD	Total	Nasal breathing Mean	SD	Total	Weight	Mean Difference IV, Random, 95% CI
D'Ascanio,2010	79.01	3.69	98	81.78	1.67	98	43.5%	-2.77 [-3.57, -1.97]
Juliano,2009	82.6	3.8	52	84.1	4.1	90	15.7%	-1.50 [-2.84, -0.16]
Juliano,2013(1)	82.1	3.8	52	83.7	4.1	92	15.8%	-1.60 [-2.93, -0.27]
Juliano,2013(2)	83	3.8	52	84.8	4	92	16.1%	-1.80 [-3.12, -0.48]
Juliano.2009	83.33	3.99	15	85.67	5.26	12	2.2%	-2.34 [-5.94, 1.26]
Mattar,2011	81.6	3.9	44	82.8	4.6	29	6.8%	-1.20 [-3.23, 0.83]
**Total (95% CI)**			**313**			**413**	**100.0%**	**-2.11 [-2.64, -1.59]**

Heterogeneity: Tau² = 0.00; Chi² = 4.97, df = 5 (P = 0.42); I² = 0%
Test for overall effect: Z = 7.84 (P < 0.00001)

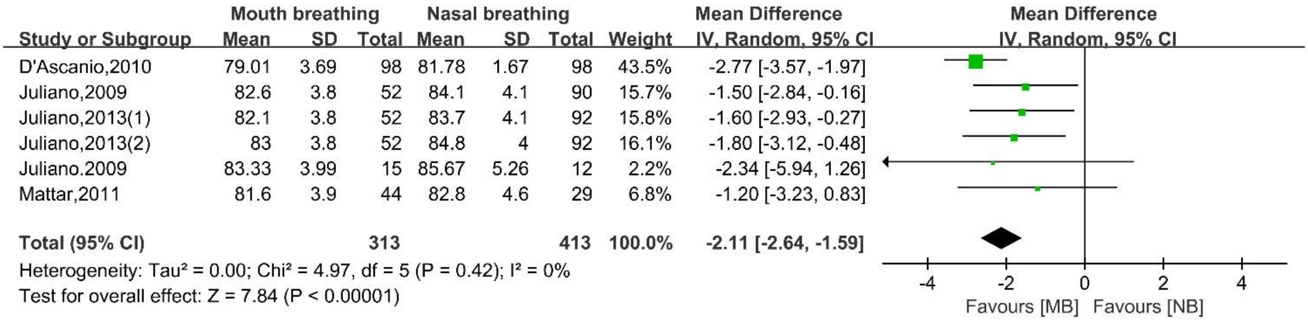

E2.3 Forest plot of SNA

Study or Subgroup	Mouth breathing Mean	SD	Total	Nasal breathing Mean	SD	Total	Weight	Mean Difference IV, Random, 95% CI
Juliano,2009	21.1	3.7	52	18.1	4.4	90	32.0%	3.00 [1.64, 4.36]
Juliano,2013(1)	21.3	3.6	52	18.4	4.2	92	34.7%	2.90 [1.60, 4.20]
Juliano,2013(2)	20.8	3.8	52	17.4	4.9	92	28.4%	3.40 [1.96, 4.84]
Juliano.2009	22	3.32	15	16.5	5.28	12	5.0%	5.50 [2.07, 8.93]
**Total (95% CI)**			**171**			**286**	**100.0%**	**3.20 [2.44, 3.97]**

Heterogeneity: Tau² = 0.00; Chi² = 2.09, df = 3 (P = 0.55); I² = 0%
Test for overall effect: Z = 8.19 (P < 0.00001)

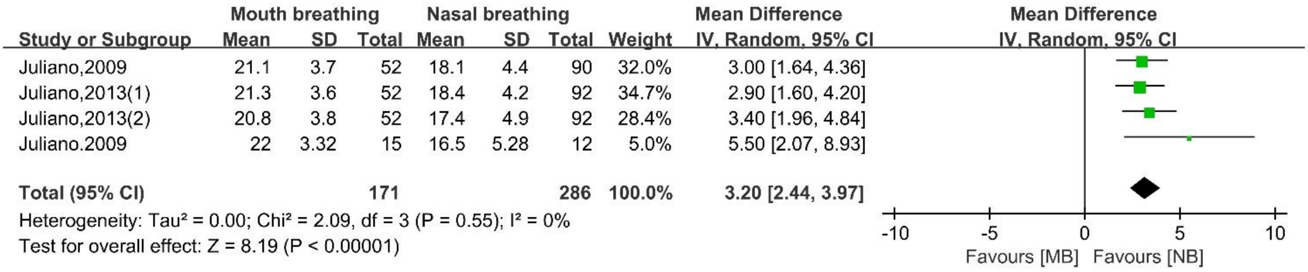

E2.4 Forest plot of SN-OP

Study or Subgroup	Mouth breathing Mean	SD	Total	Nasal breathing Mean	SD	Total	Weight	Mean Difference IV, Random, 95% CI
D'Ascanio,2010	16.47	1.58	98	15.74	1.93	98	91.9%	0.73 [0.24, 1.22]
Mattar,2011	6.1	3.1	44	6	3.8	29	8.1%	0.10 [-1.56, 1.76]
**Total (95% CI)**			**142**			**127**	**100.0%**	**0.68 [0.21, 1.15]**

Heterogeneity: Tau² = 0.00; Chi² = 0.51, df = 1 (P = 0.48); I² = 0%
Test for overall effect: Z = 2.81 (P = 0.005)

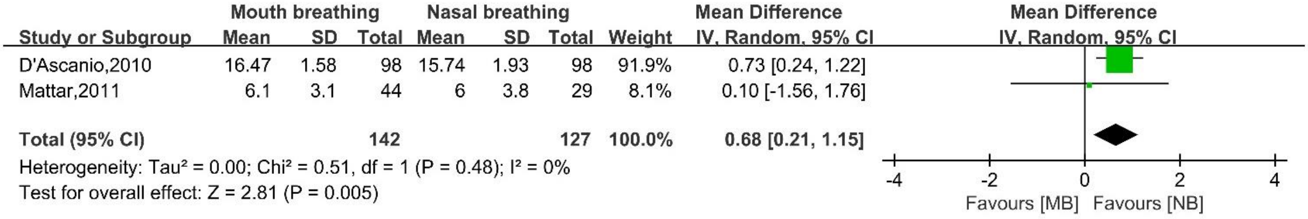

E2.5 Forest plot of SN-PP

Study or Subgroup	Mouth breathing Mean	SD	Total	Nasal breathing Mean	SD	Total	Weight	Mean Difference IV, Random, 95% CI
D'Ascanio,2010	30.73	3.37	98	25.76	2.84	98	89.2%	4.97 [4.10, 5.84]
Mattar,2011	37.2	4.2	44	32.7	6	29	10.8%	4.50 [1.99, 7.01]
**Total (95% CI)**			**142**			**127**	**100.0%**	**4.92 [4.10, 5.74]**

Heterogeneity: Tau² = 0.00; Chi² = 0.12, df = 1 (P = 0.73); I² = 0%
Test for overall effect: Z = 11.70 (P < 0.00001)

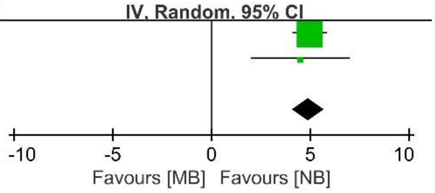

E2.6 Forest plot of PP-MP

Study or Subgroup	Mouth breathing Mean	SD	Total	Nasal breathing Mean	SD	Total	Weight	Mean Difference IV, Random, 95% CI
Juliano,2009	38.3	5.2	52	33.5	5.7	90	25.4%	4.80 [2.96, 6.64]
Juliano,2013(1)	38	4.6	52	34.3	5.5	92	28.2%	3.70 [2.02, 5.38]
Juliano,2013(2)	38.5	6	52	32.1	6.3	92	21.7%	6.40 [4.32, 8.48]
Juliano.2009	38.53	5.63	15	30.25	7.21	12	5.2%	8.28 [3.30, 13.26]
Mattar,2011	41.2	4.8	44	36.6	4.8	29	19.5%	4.60 [2.35, 6.85]
**Total (95% CI)**			**215**			**315**	**100.0%**	**4.98 [3.80, 6.16]**

Heterogeneity: Tau² = 0.56; Chi² = 5.81, df = 4 (P = 0.21); I² = 31%
Test for overall effect: Z = 8.24 (P < 0.00001)

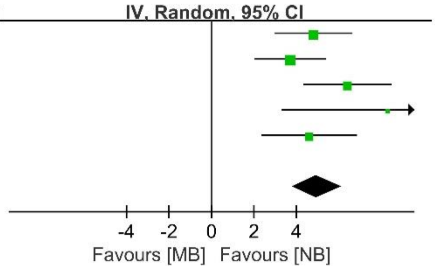

E2.7 Forest plot of SNGoGn

Study or Subgroup	Mouth breathing Mean	SD	Total	Nasal breathing Mean	SD	Total	Weight	Mean Difference IV, Random, 95% CI
Juliano,2009	5.3	2.3	52	4.5	1.9	90	30.4%	0.80 [0.06, 1.54]
Juliano,2013(1)	5.7	2	52	4.7	2	92	34.0%	1.00 [0.32, 1.68]
Juliano,2013(2)	4.8	2.6	52	4.1	1.6	92	28.1%	0.70 [-0.08, 1.48]
Juliano.2009	4.4	2.72	15	5.25	1.76	12	7.5%	-0.85 [-2.55, 0.85]
**Total (95% CI)**			**171**			**286**	**100.0%**	**0.72 [0.23, 1.20]**

Heterogeneity: Tau² = 0.06; Chi² = 3.96, df = 3 (P = 0.27); I² = 24%
Test for overall effect: Z = 2.89 (P = 0.004)

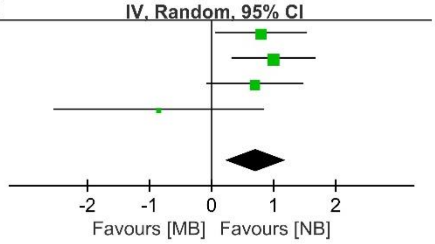

E2.8 Forest plot of 1-NA

Study or Subgroup	Mouth breathing Mean	SD	Total	Nasal breathing Mean	SD	Total	Weight	Mean Difference IV, Random, 95% CI
Juliano,2009	26.3	6.2	52	24.1	7.7	90	27.5%	2.20 [-0.12, 4.52]
Juliano,2013(1)	27.3	5.4	52	23.7	5.7	92	33.6%	3.60 [1.73, 5.47]
Juliano,2013(2)	25.3	7	52	24.1	6.3	92	27.7%	1.20 [-1.10, 3.50]
Juliano.2009	25.13	6.91	15	26.58	4.98	12	11.2%	-1.45 [-5.94, 3.04]
**Total (95% CI)**			**171**			**286**	**100.0%**	**1.98 [0.30, 3.66]**

Heterogeneity: Tau² = 1.27; Chi² = 5.41, df = 3 (P = 0.14); I² = 45%
Test for overall effect: Z = 2.32 (P = 0.02)

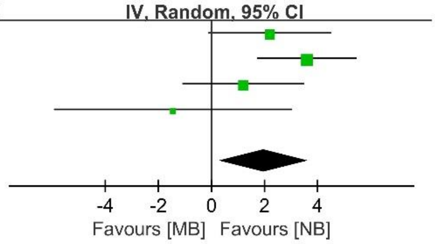

E2.9 Forest plot of 1.NA

Study or Subgroup	Mouth breathing Mean	SD	Total	Nasal breathing Mean	SD	Total	Weight	Mean Difference IV, Random, 95% CI
Juliano,2009	7.5	2.8	52	6.4	2.3	90	32.6%	1.10 [0.20, 2.00]
Juliano,2013(1)	7.7	3	52	6.6	2.4	92	29.0%	1.10 [0.15, 2.05]
Juliano,2013(2)	7.2	2.6	52	6.2	2.1	92	38.4%	1.00 [0.17, 1.83]
**Total (95% CI)**			**156**			**274**	**100.0%**	**1.06 [0.55, 1.57]**

Heterogeneity: Tau² = 0.00; Chi² = 0.03, df = 2 (P = 0.98); I² = 0%
Test for overall effect: Z = 4.06 (P < 0.0001)

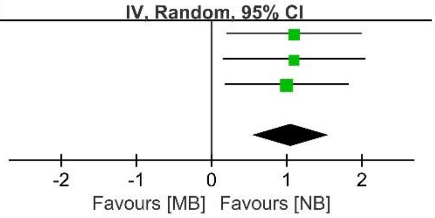

E2.10 Forest plot of 1-NB

Study or Subgroup	MB Mean	SD	Total	NB Mean	SD	Total	Weight	Mean Difference IV, Random, 95% CI
Juliano,2009	31.8	5.8	52	30.9	5.4	90	30.1%	0.90 [-1.03, 2.83]
Juliano,2013(1)	33	6.1	52	30.9	5.7	92	28.9%	2.10 [0.07, 4.13]
Juliano,2013(2)	30.4	5.1	52	31.1	5.1	92	32.8%	-0.70 [-2.43, 1.03]
Juliano.2009	30.27	7.57	15	33.25	6.34	12	8.2%	-2.98 [-8.23, 2.27]
**Total (95% CI)**			**171**			**286**	**100.0%**	**0.41 [-1.23, 2.04]**

Heterogeneity: Tau² = 1.33; Chi² = 6.08, df = 3 (P = 0.11); I² = 51%
Test for overall effect: Z = 0.49 (P = 0.63)

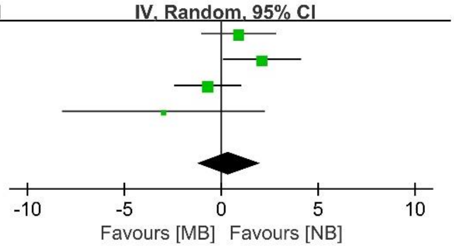

E2.11 Forest plot of 1.NB

Study or Subgroup	Mouth breathing Mean	SD	Total	Nasal breathing Mean	SD	Total	Weight	Mean Difference IV, Random, 95% CI
Juliano,2009	4.9	1.9	52	10.3	2.8	90	26.3%	-5.40 [-6.18, -4.62]
Juliano,2013(1)	4.6	2	52	10.4	3.1	92	26.1%	-5.80 [-6.63, -4.97]
Juliano,2013(2)	5.2	1.7	52	9.8	2.2	92	26.6%	-4.60 [-5.24, -3.96]
Juliano.2009	4.47	1.68	15	1.25	3.47	12	21.0%	3.22 [1.08, 5.36]
**Total (95% CI)**			**171**			**286**	**100.0%**	**-3.48 [-5.52, -1.43]**

Heterogeneity: Tau² = 3.99; Chi² = 62.14, df = 3 (P < 0.00001); I² = 95%
Test for overall effect: Z = 3.33 (P = 0.0009)

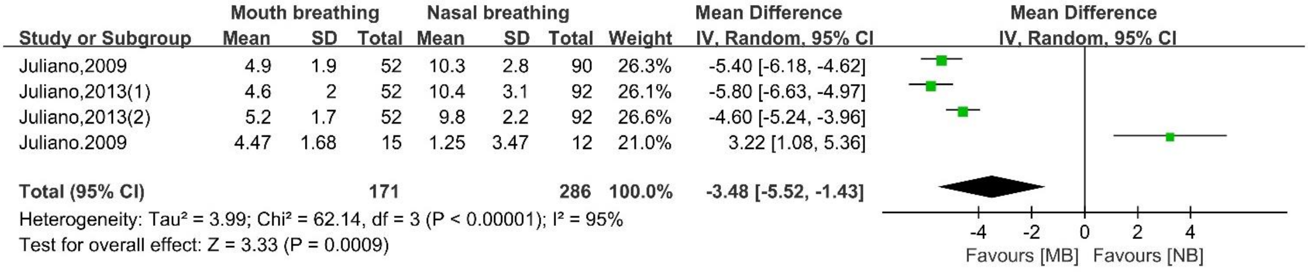

E2.12 Forest plot of SPAS

Study or Subgroup	Mouth breathing Mean	SD	Total	Nasal breathing Mean	SD	Total	Weight	Mean Difference IV, Random, 95% CI
Juliano,2009	10.1	3.1	52	12.3	3.1	90	29.0%	-2.20 [-3.26, -1.14]
Juliano,2013(1)	9.7	3.6	52	12.6	3.3	92	25.5%	-2.90 [-4.09, -1.71]
Juliano,2013(2)	10.5	2.4	52	11.8	2.8	92	35.0%	-1.30 [-2.17, -0.43]
Juliano.2009	9.93	2.84	15	12.58	2.97	12	10.5%	-2.65 [-4.86, -0.44]
**Total (95% CI)**			**171**			**286**	**100.0%**	**-2.11 [-2.90, -1.32]**

Heterogeneity: Tau² = 0.26; Chi² = 5.17, df = 3 (P = 0.16); I² = 42%
Test for overall effect: Z = 5.26 (P < 0.00001)

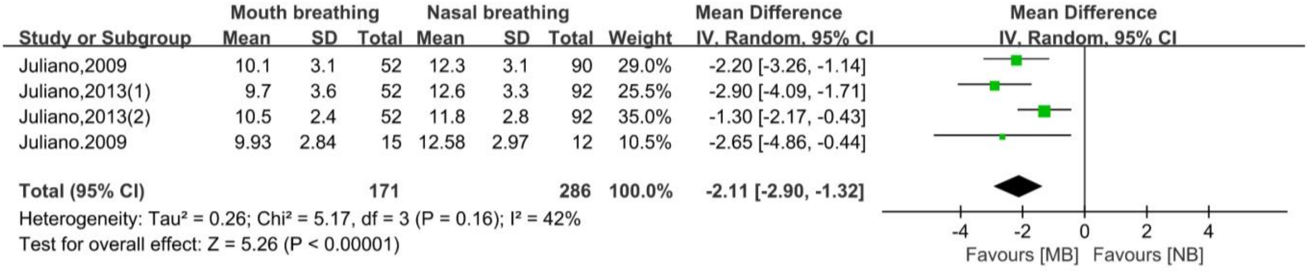

E2.13 Forest plot of PAS

Study or Subgroup	Mouth breathing Mean	SD	Total	Nasal breathing Mean	SD	Total	Weight	Mean Difference IV, Random, 95% CI
Juliano,2009	33.6	3.2	52	34.9	3.4	90	30.6%	-1.30 [-2.42, -0.18]
Juliano,2013(1)	33.4	2.6	52	34.7	3.4	92	39.0%	-1.30 [-2.29, -0.31]
Juliano,2013(2)	33.8	3.8	52	35.1	3.4	92	24.7%	-1.30 [-2.54, -0.06]
Juliano.2009	32.27	2.6	15	34.33	3.94	12	5.7%	-2.06 [-4.65, 0.53]
**Total (95% CI)**			**171**			**286**	**100.0%**	**-1.34 [-1.96, -0.72]**

Heterogeneity: Tau² = 0.00; Chi² = 0.31, df = 3 (P = 0.96); I² = 0%
Test for overall effect: Z = 4.26 (P < 0.0001)

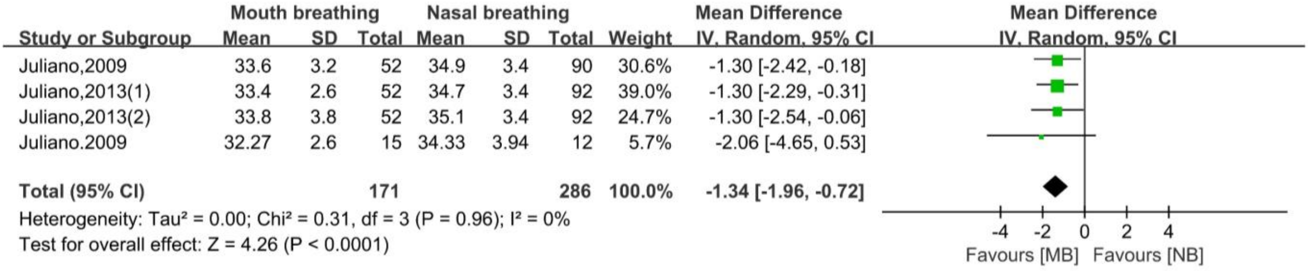

E2.14 Forest plot of C3-H

Study or Subgroup	Mouth breathing Mean	SD	Total	Nasal breathing Mean	SD	Total	Weight	Mean Difference IV, Random, 95% CI
Juliano,2009	11.8	5.3	52	11.3	5.2	90	31.1%	0.50 [-1.30, 2.30]
Juliano,2013(1)	12.1	4.7	52	10.9	5	92	36.0%	1.20 [-0.44, 2.84]
Juliano,2013(2)	11.5	6	52	12.4	5.1	92	27.7%	-0.90 [-2.84, 1.04]
Juliano.2009	14.4	5.17	15	11.58	7.25	12	5.2%	2.82 [-2.05, 7.69]
**Total (95% CI)**			**171**			**286**	**100.0%**	**0.49 [-0.64, 1.61]**

Heterogeneity: Tau² = 0.22; Chi² = 3.59, df = 3 (P = 0.31); I² = 16%
Test for overall effect: Z = 0.84 (P = 0.40)

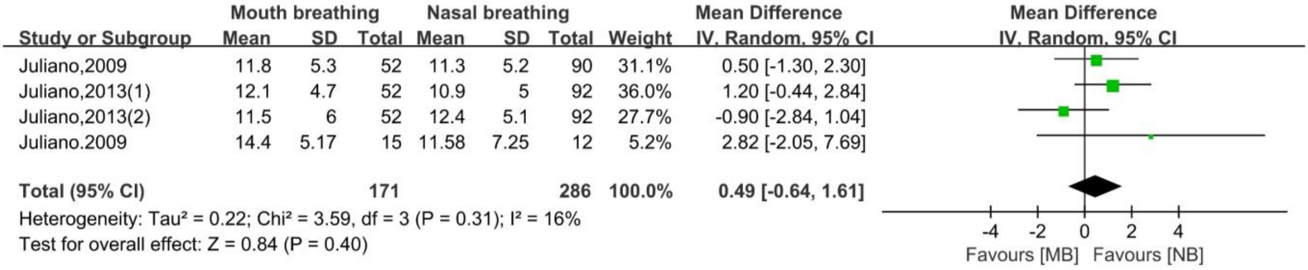

E2.15 Forest plot of MP-H
